# Supplementary figures and images for: Aberrant association of chromatin with nuclear periphery induced by Rif1 leads to mitotic defect
Source: Life Sci Alliance. 2023 Feb 7;6(4):e202201603. doi: 10.26508/lsa.202201603 (PMC9909590; doi:10.26508/lsa.202201603)

Figure 1A Upper

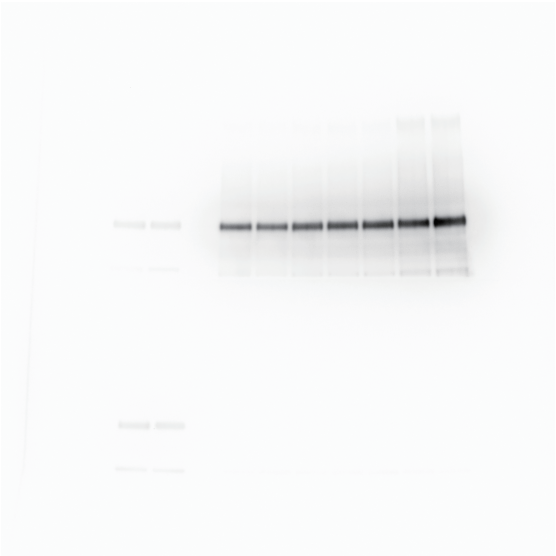

Figure 1A Lower

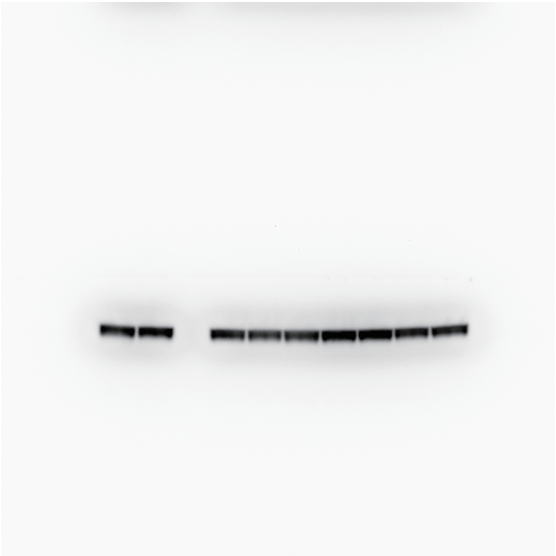

Figure 1D

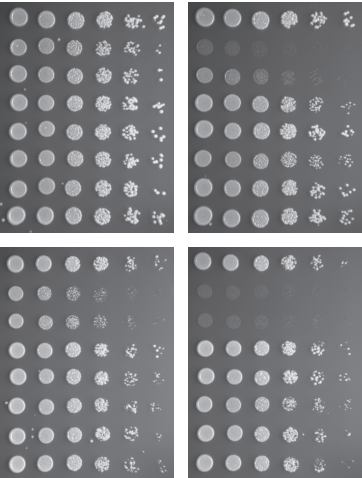

Figure 1C

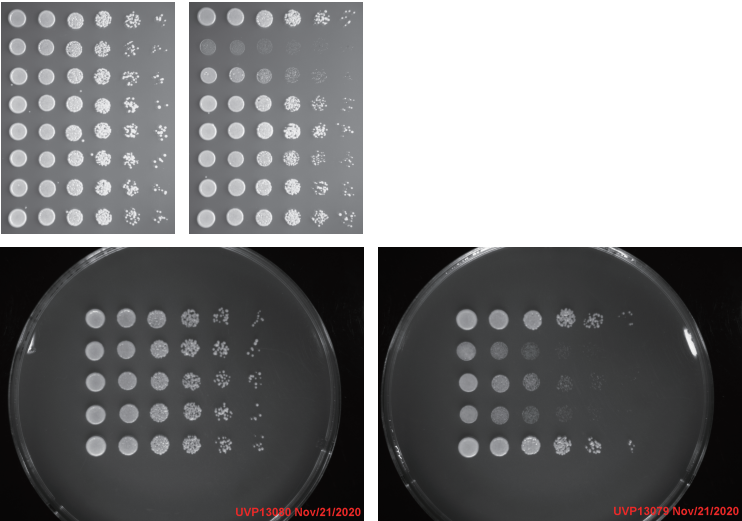

Supplement: Supplementary file 1 [file LSA-2022-01603_SdataF1.pdf]

Supplementary Figure 1A

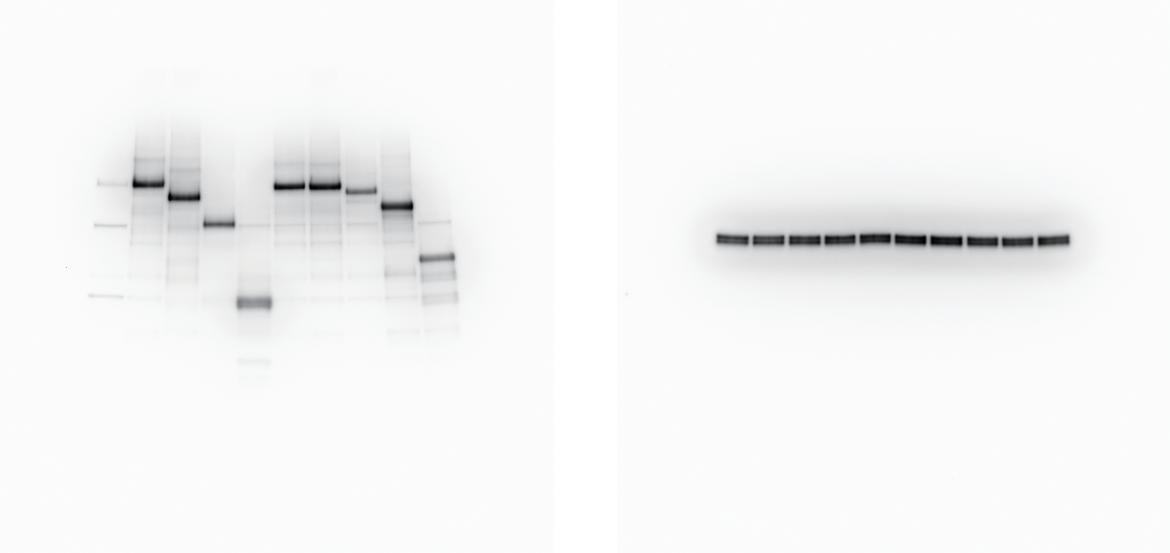

Supplementary Figure 1B-a

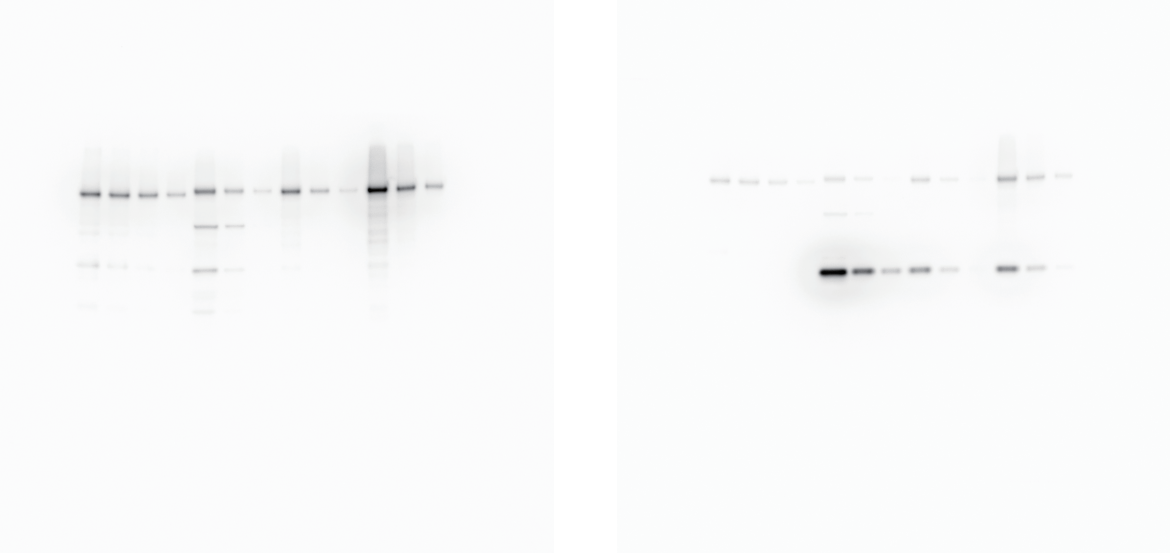

Supplementary Figure 1B-d

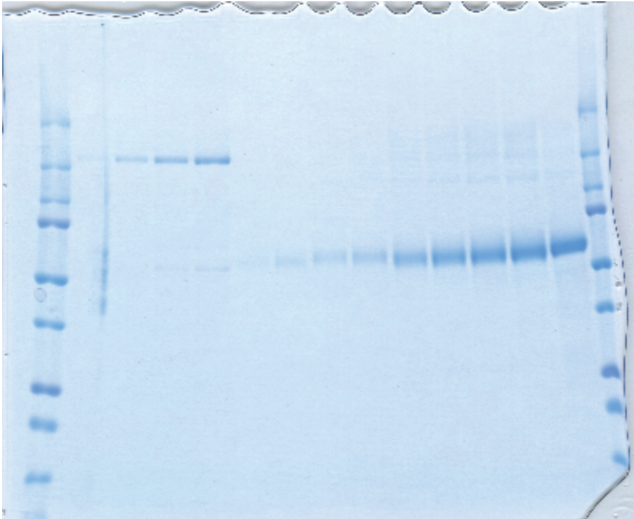

Supplement: Supplementary file 2 [file LSA-2022-01603_SdataFS1.pdf]

Figure 2A

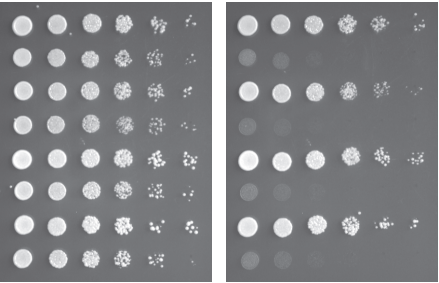

Figure 2E

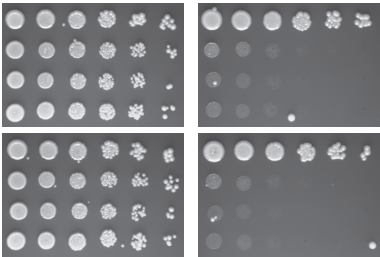

Figure 2C

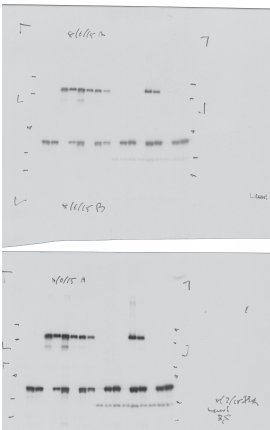

Figure 2D Upper

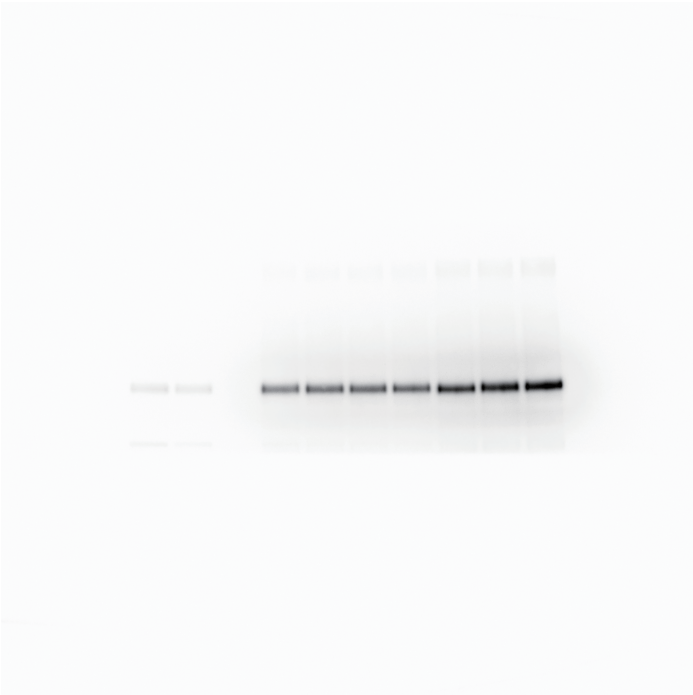

Figure 2D Lower

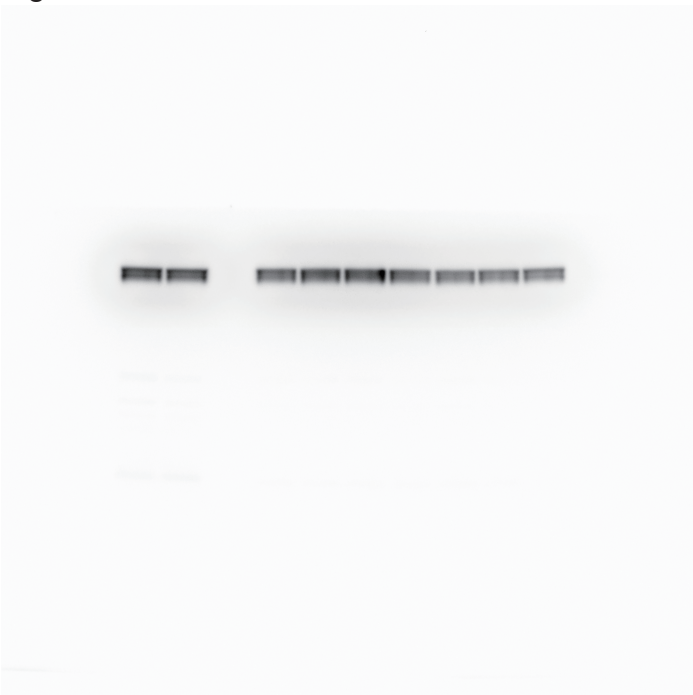

Supplement: Supplementary file 3 [file LSA-2022-01603_SdataF2.pdf]

Supplementary Figure 2A

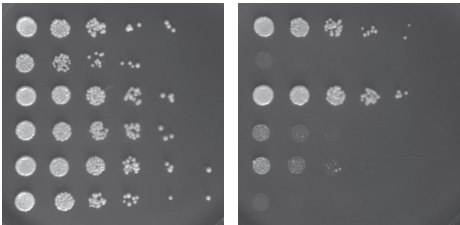

Supplementary Figure 2B

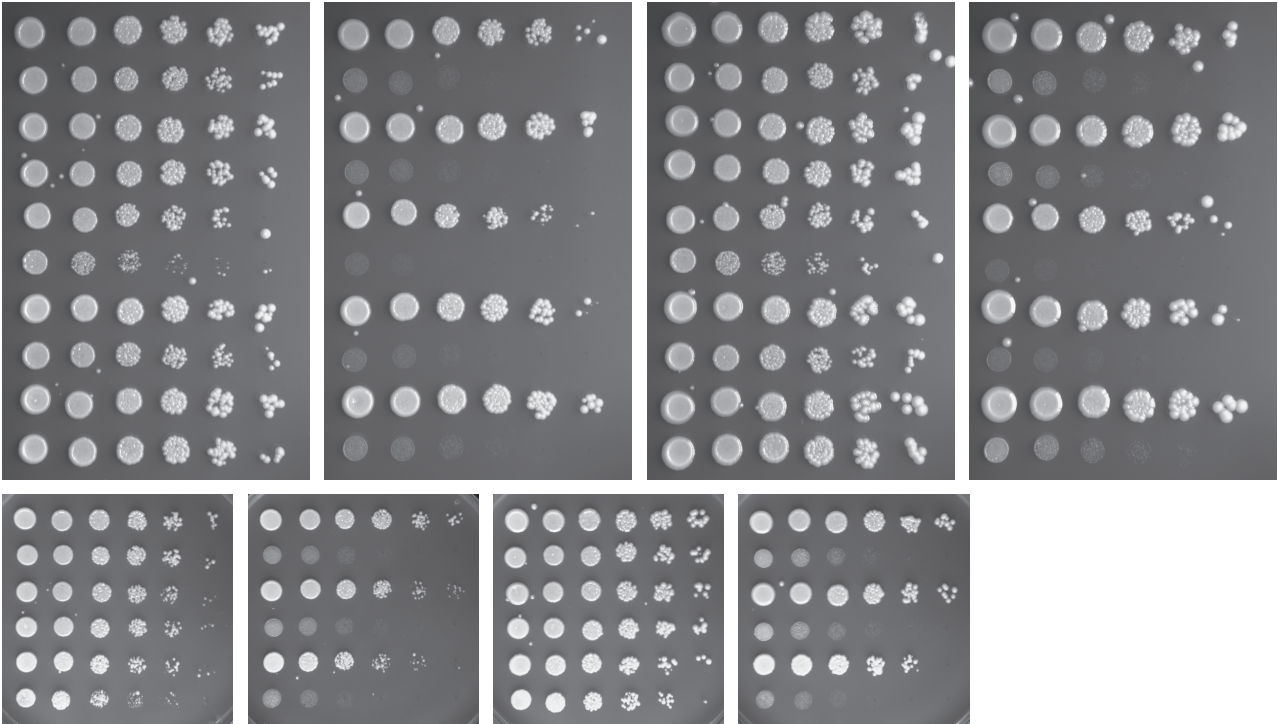

Supplementary Figure 2C

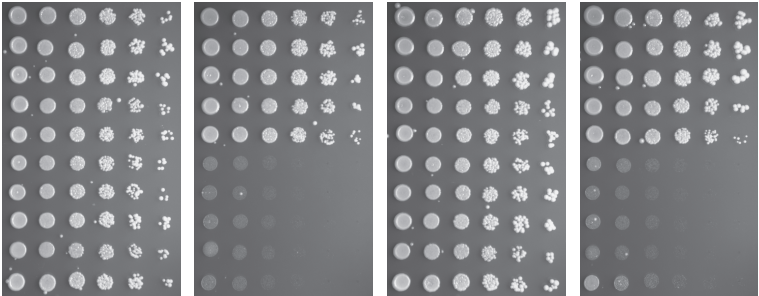

Supplement: Supplementary file 4 [file LSA-2022-01603_SdataFS2.pdf]

Figure 3B Upper

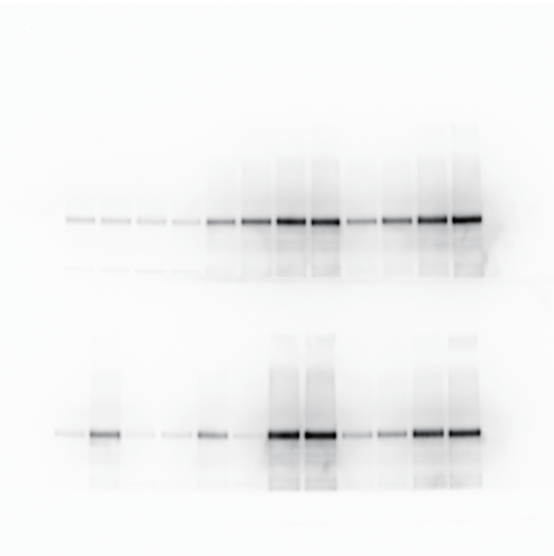

Figure 3B Lower

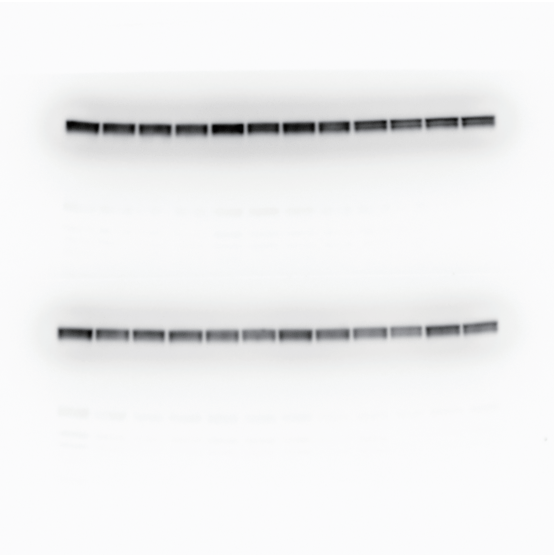

Figure 3D

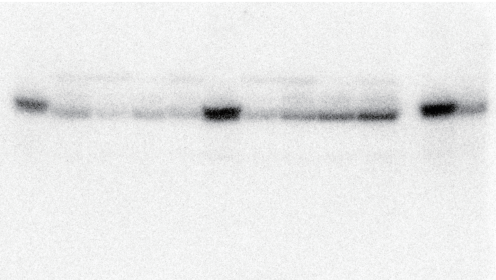

Supplement: Supplementary file 5 [file LSA-2022-01603_SdataF3.pdf]

Figure 4A

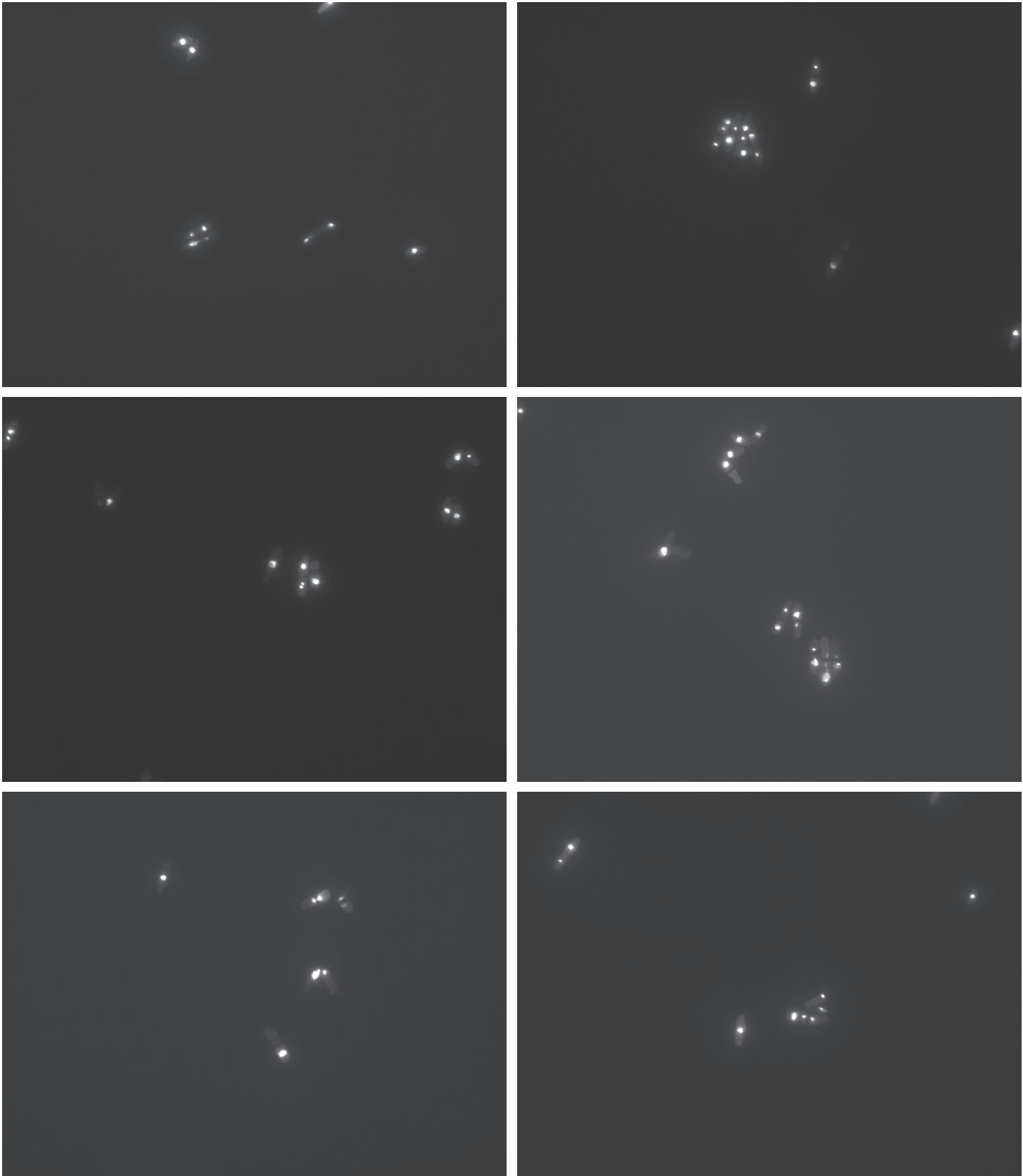

Figure 4A

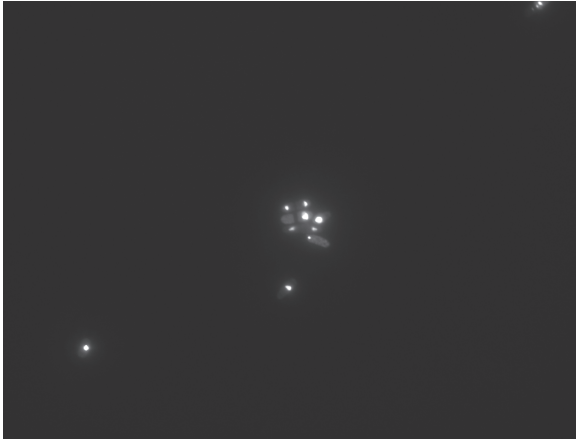

Figure 4B

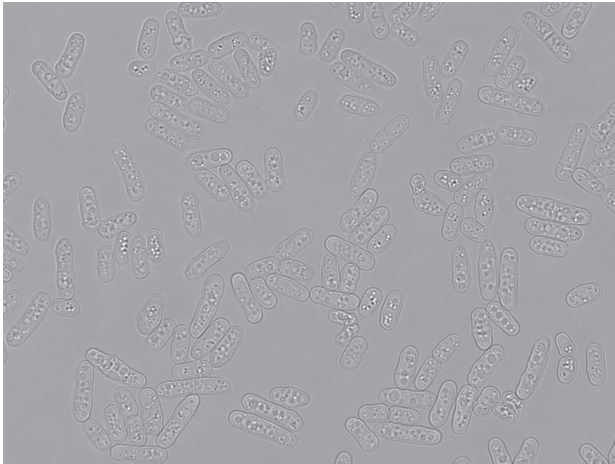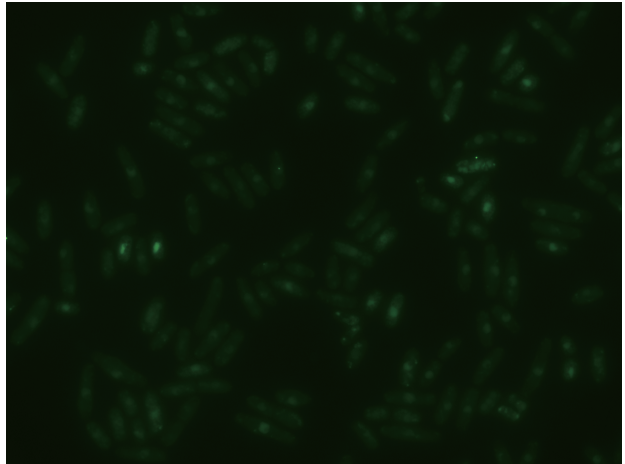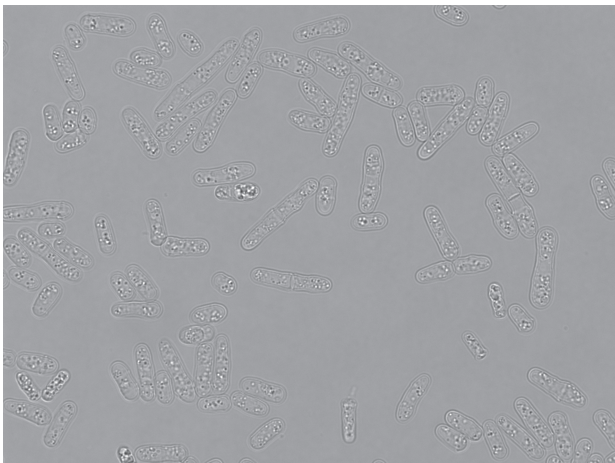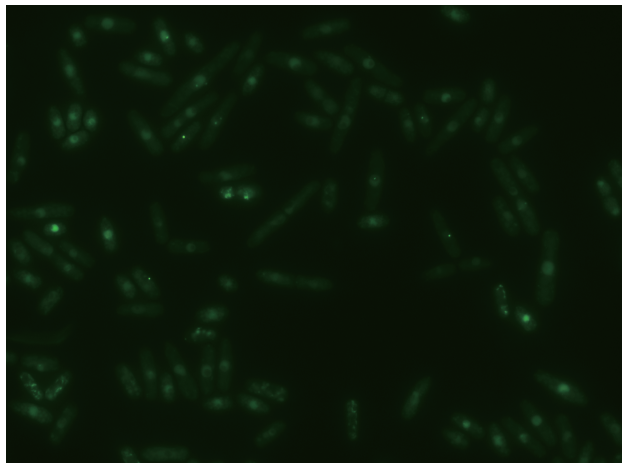

Supplement: Supplementary file 6 [file LSA-2022-01603_SdataF4.pdf]

Supplementary Figure 3B

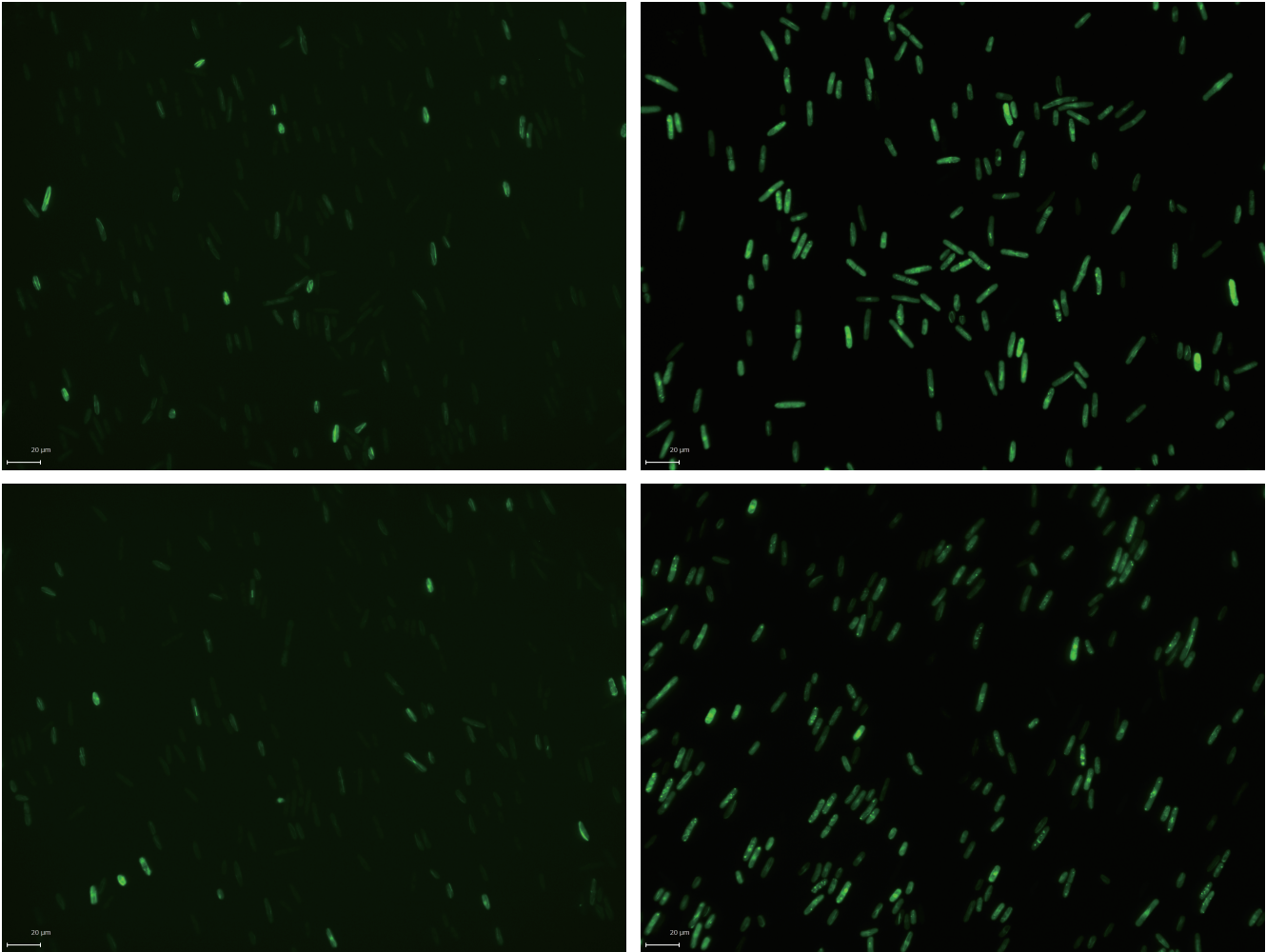

Supplement: Supplementary file 8 [file LSA-2022-01603_SdataFS3.pdf]

Figure 6A

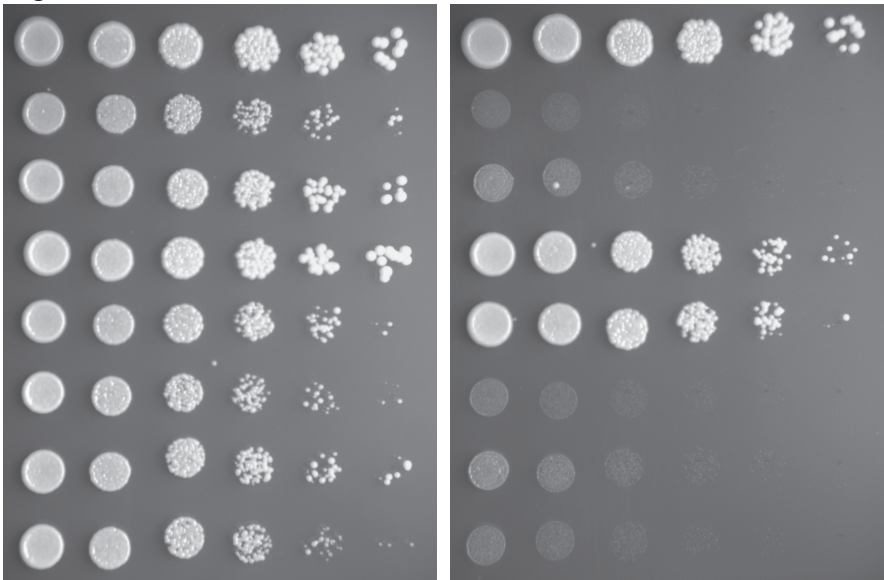

Supplement: Supplementary file 9 [file LSA-2022-01603_SdataF6.pdf]

Figure 7A 10 hr

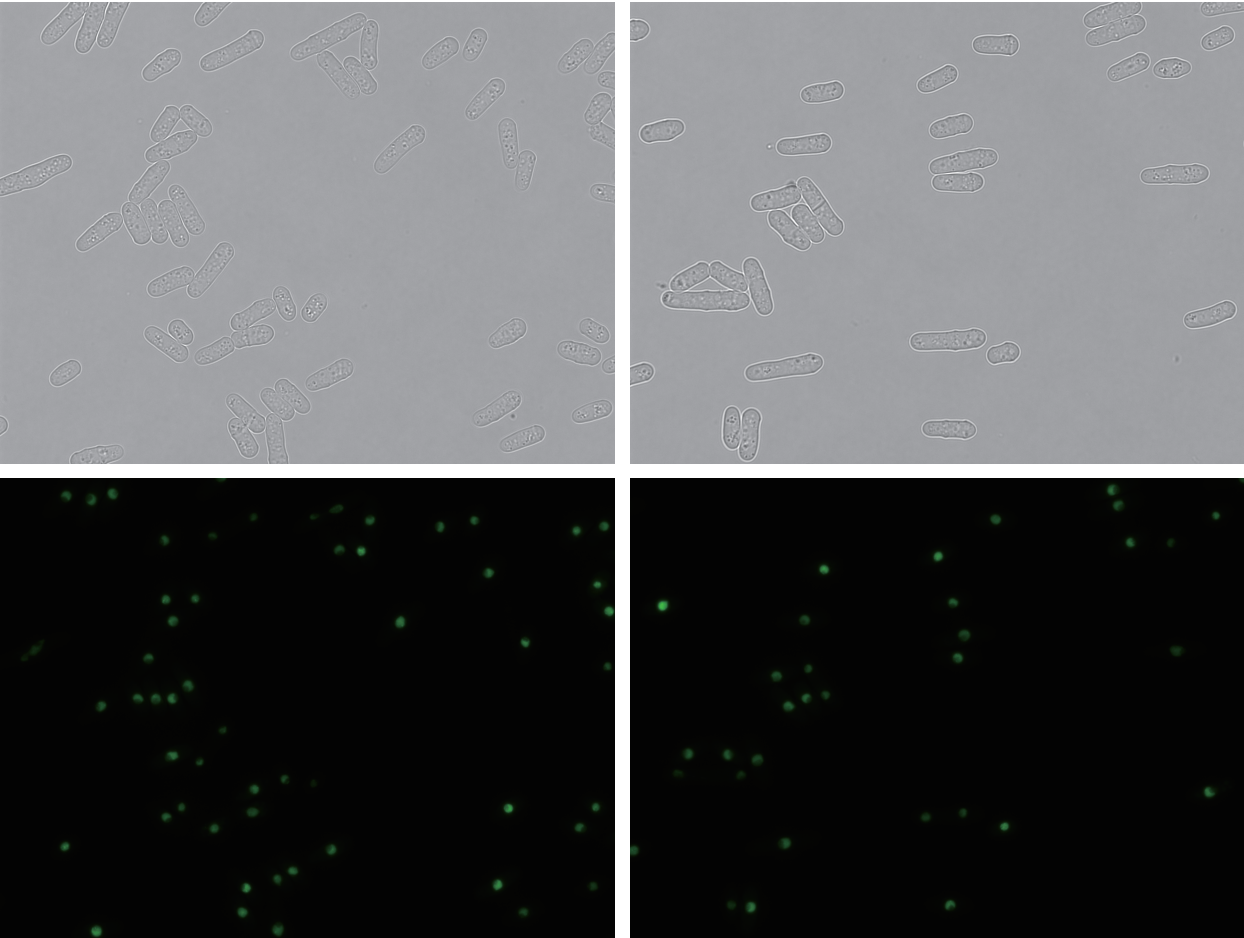

Figure 7A 18 hr

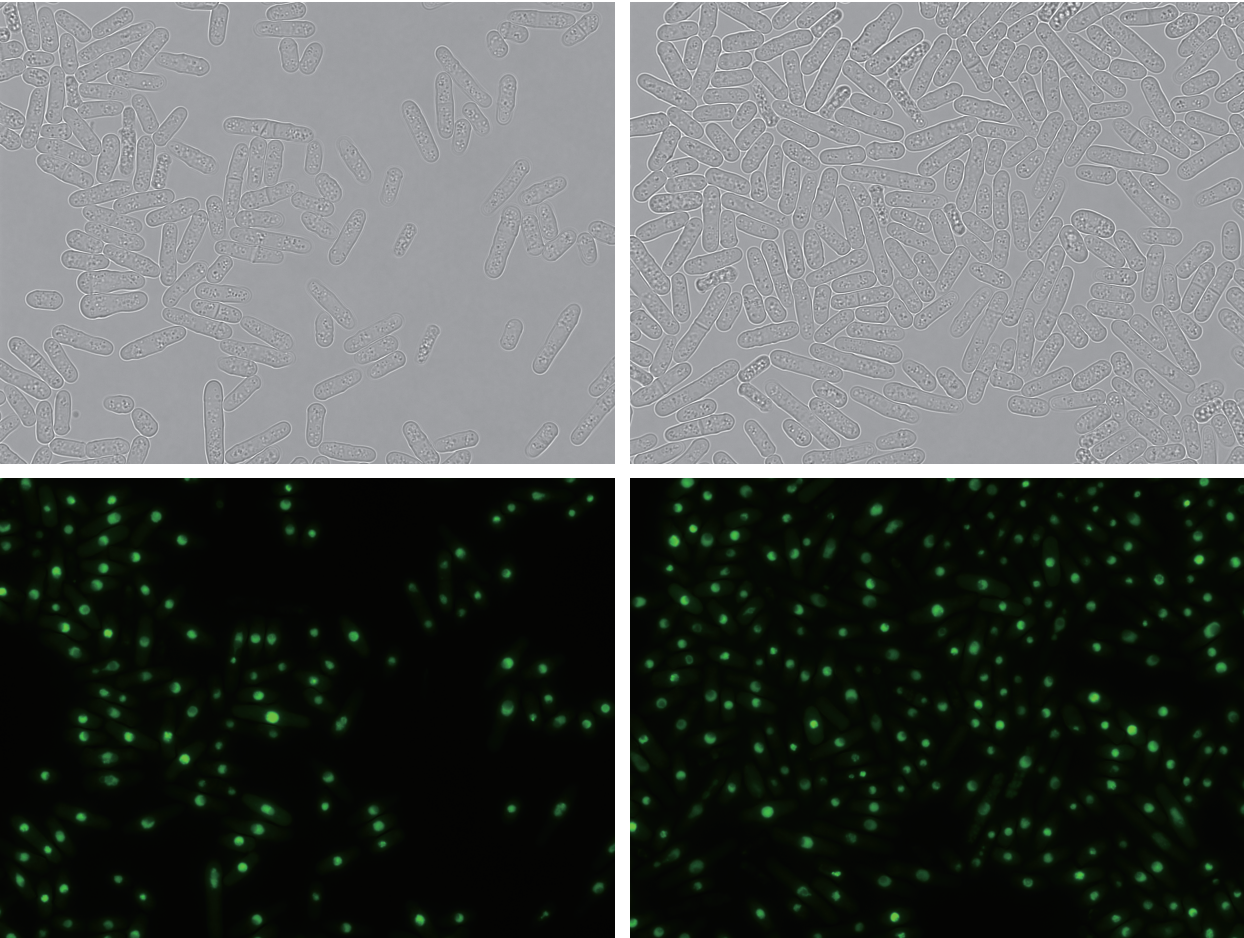

Figure 7C

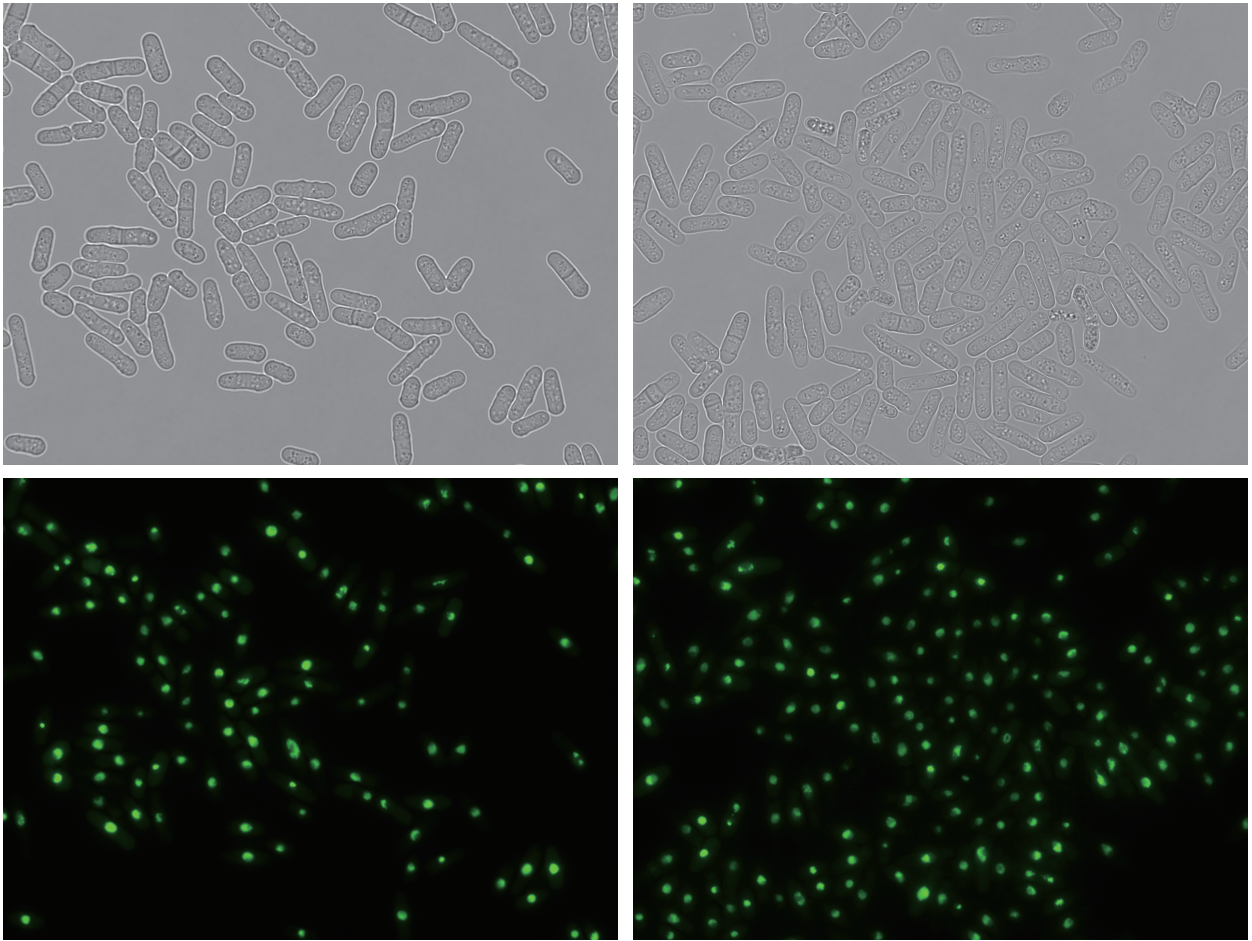

Supplement: Supplementary file 10 [file LSA-2022-01603_SdataF7.pdf]

Supplementary Figure 4A

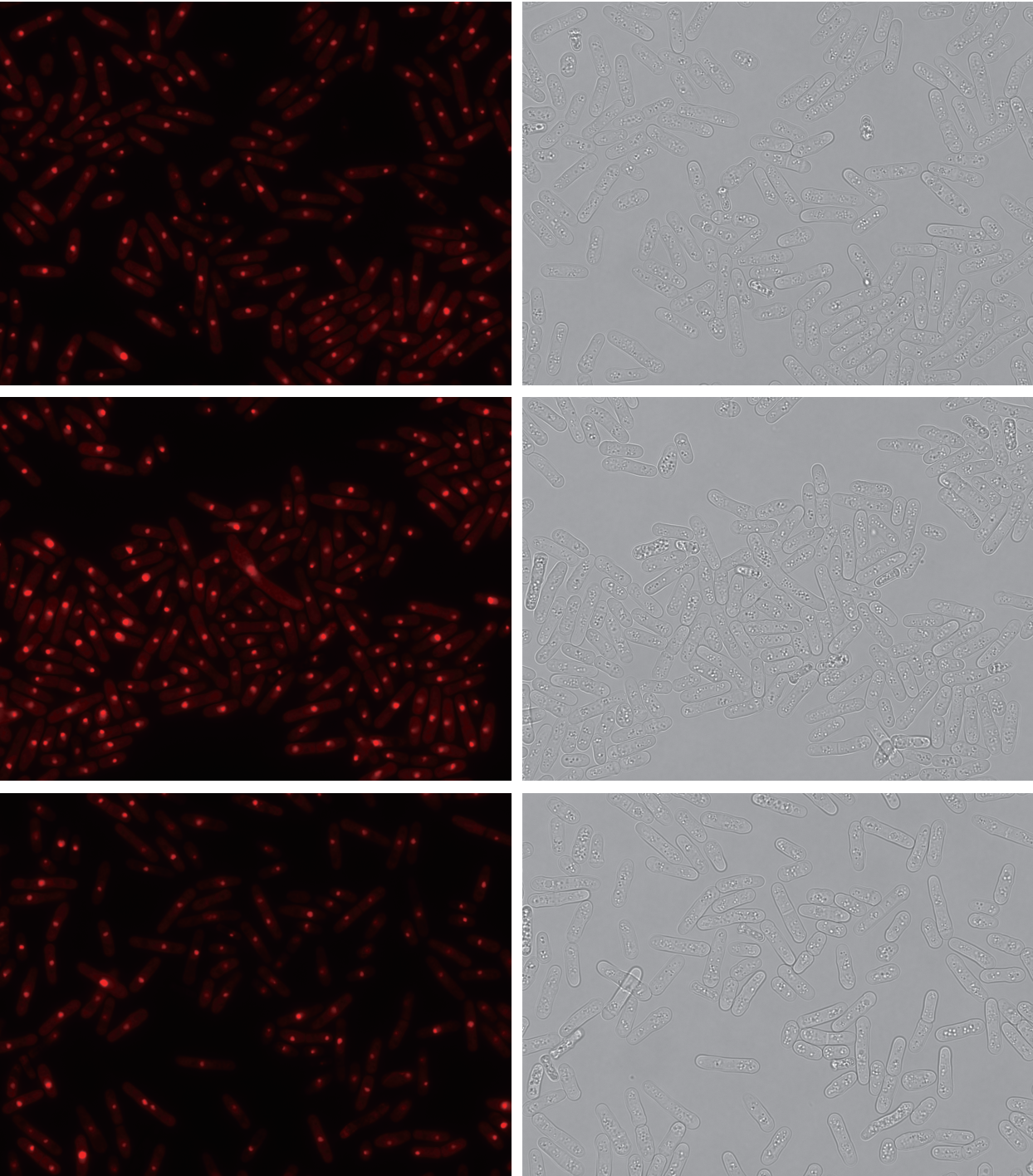

Supplement: Supplementary file 11 [file LSA-2022-01603_SdataFS4.pdf]

Figure 8A

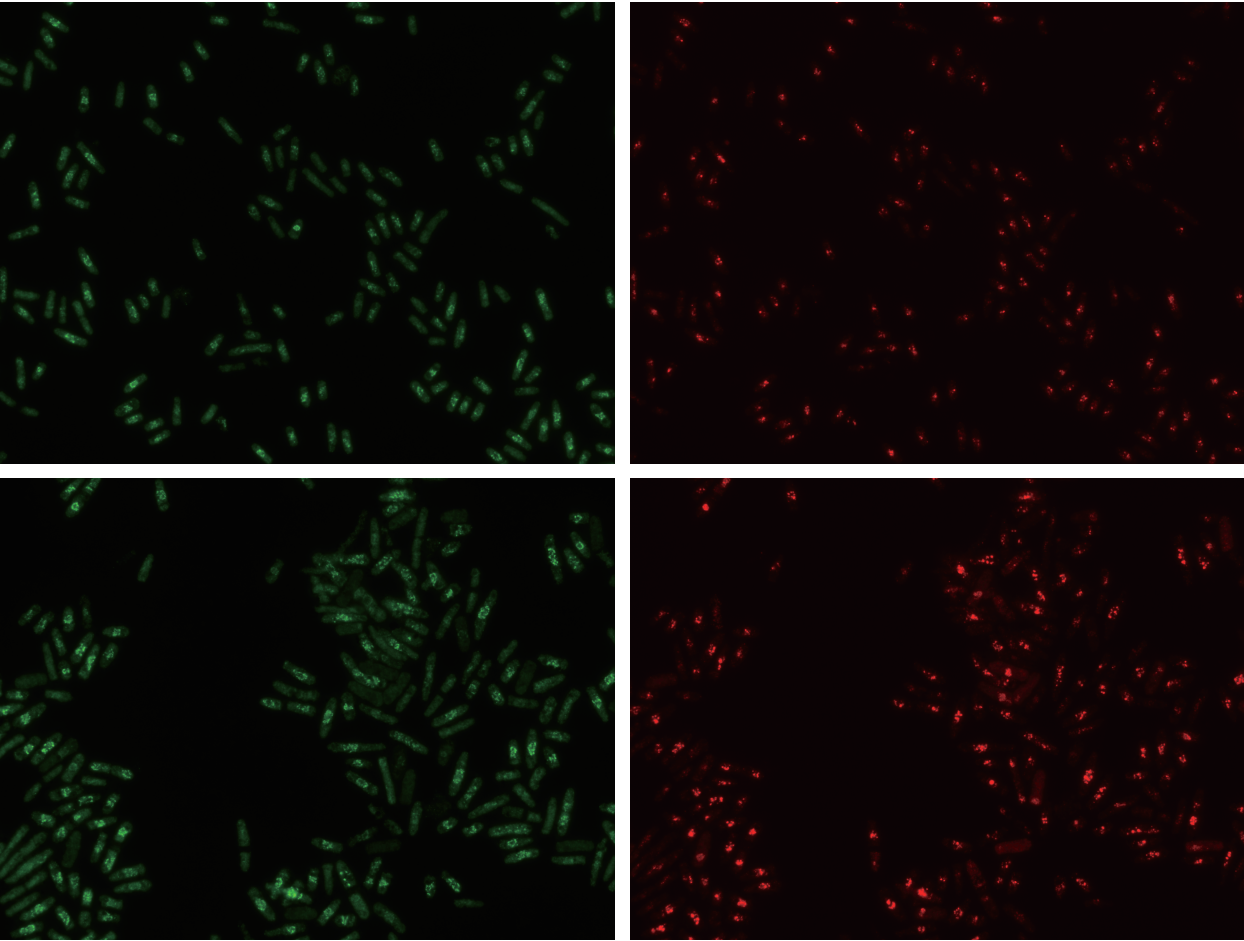

Supplement: Supplementary file 18 [file LSA-2022-01603_SdataF8.pdf]
