# Supplementary material for: Aberrant association of chromatin with nuclear periphery induced by Rif1 leads to mitotic defect
Source: Life Sci Alliance. 2023 Feb 7;6(4):e202201603. doi: 10.26508/lsa.202201603 (PMC9909590; doi:10.26508/lsa.202201603)

Kanoh *et al.* SourceData Figure 5

Figure 5A

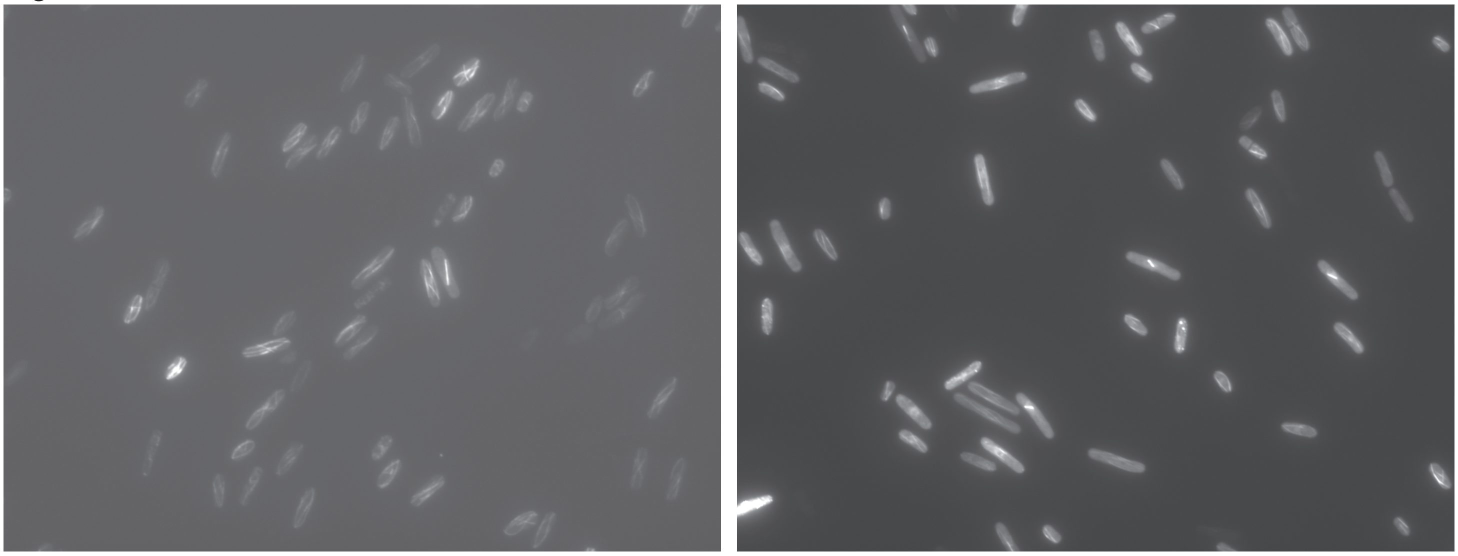

Figure 5C Vector

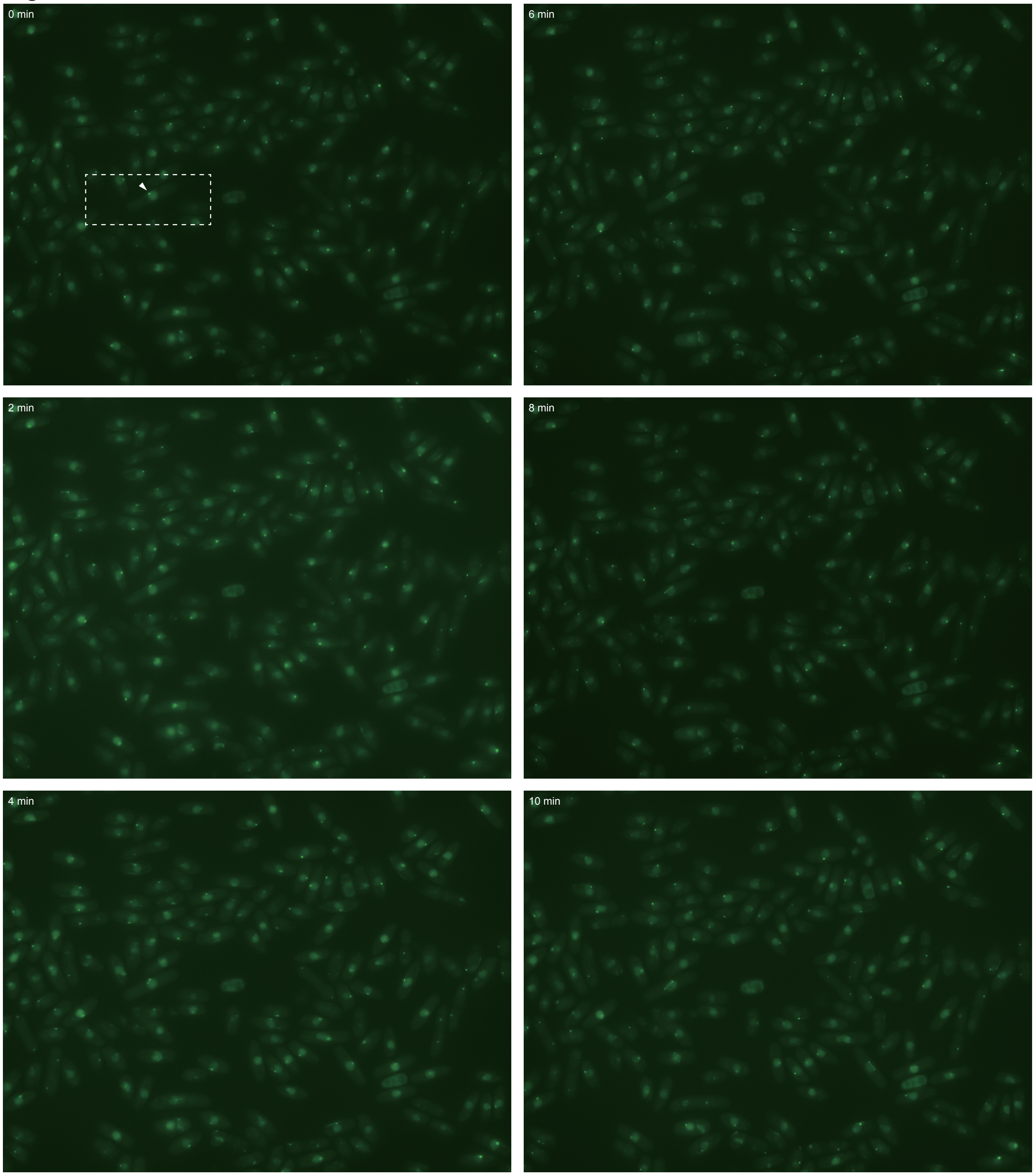

Figure 5C Vector

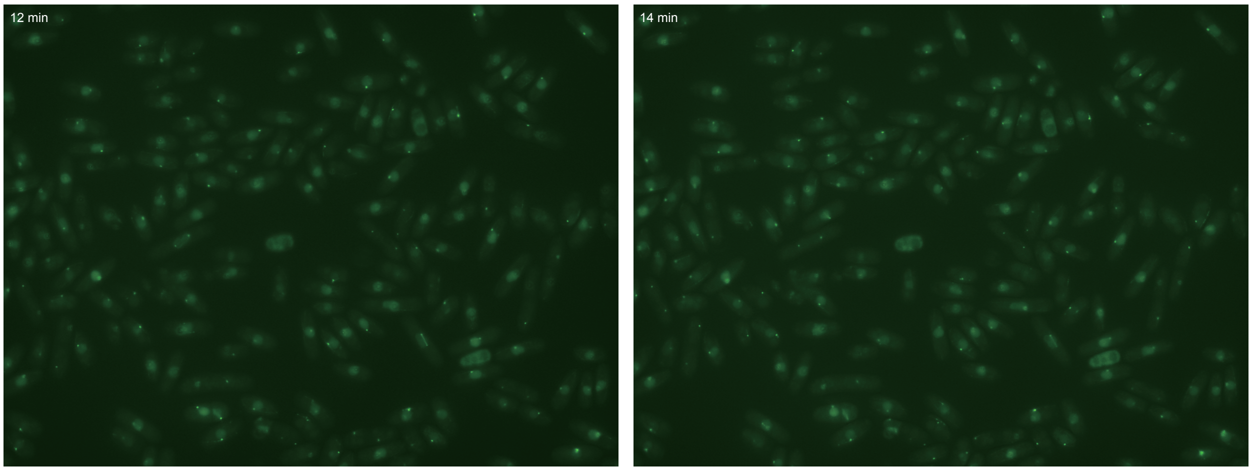

Figure 5D Rif1 OE

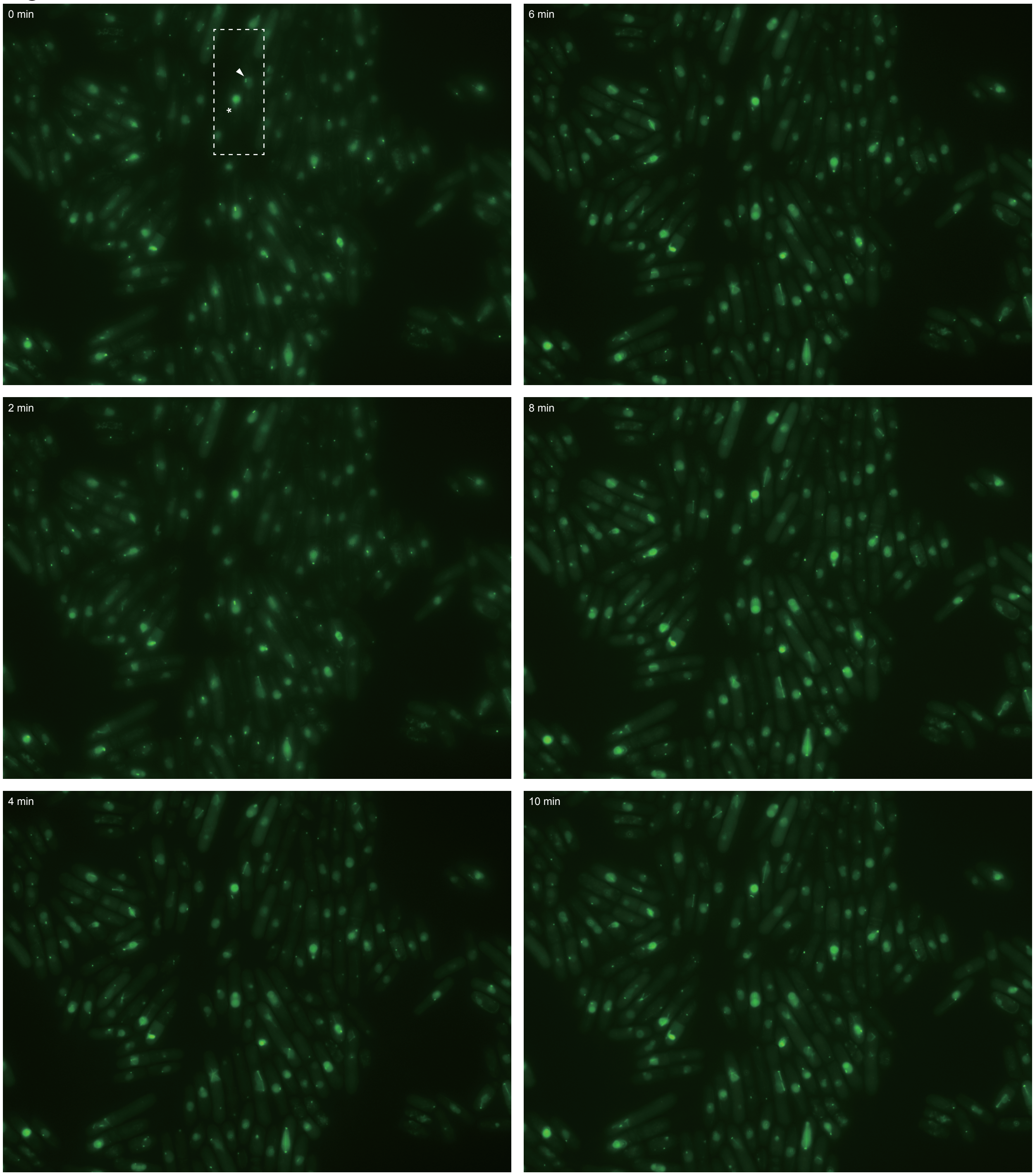

Figure 5D Rif1 OE

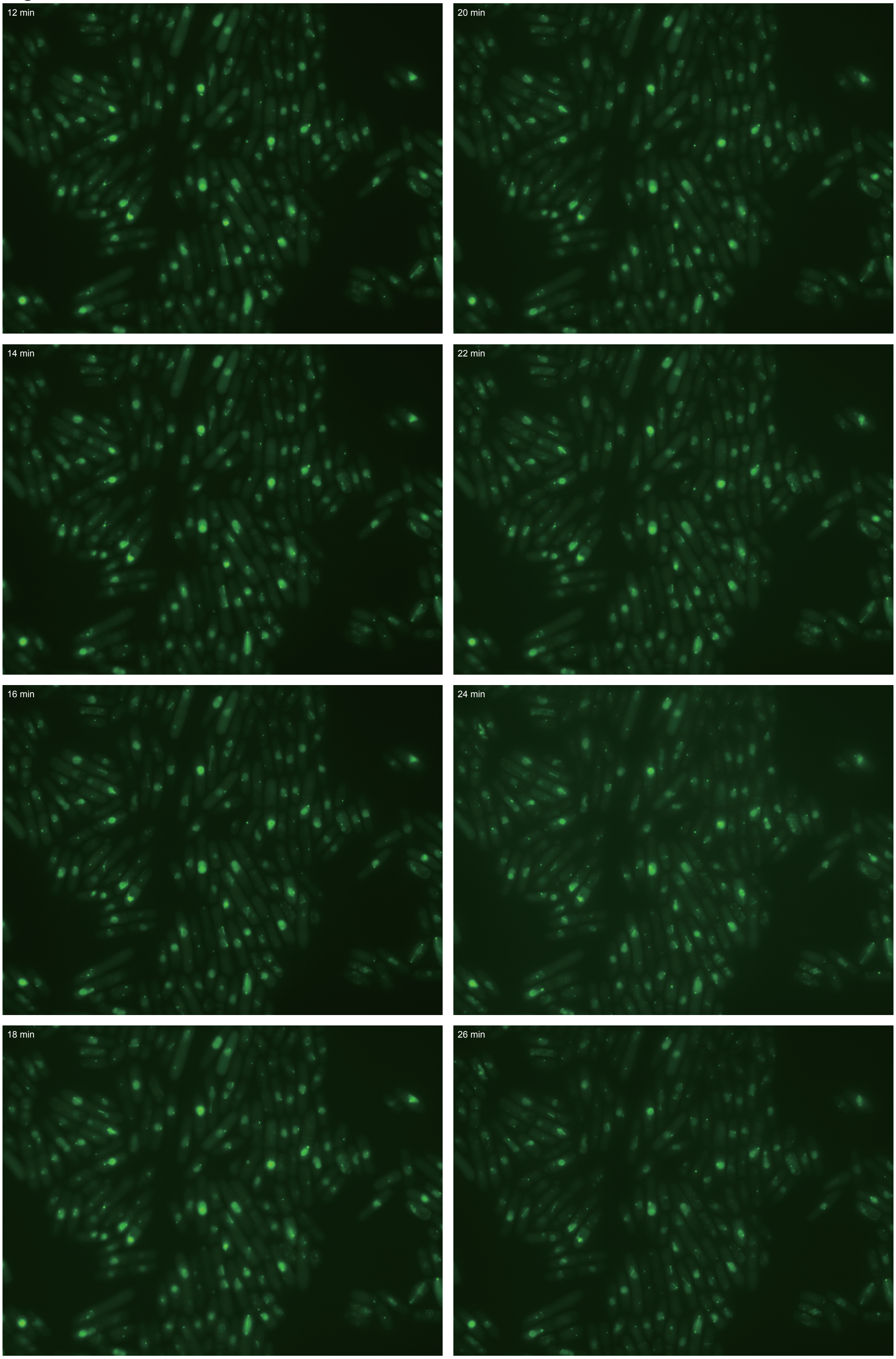

Figure 5D Rif1 OE

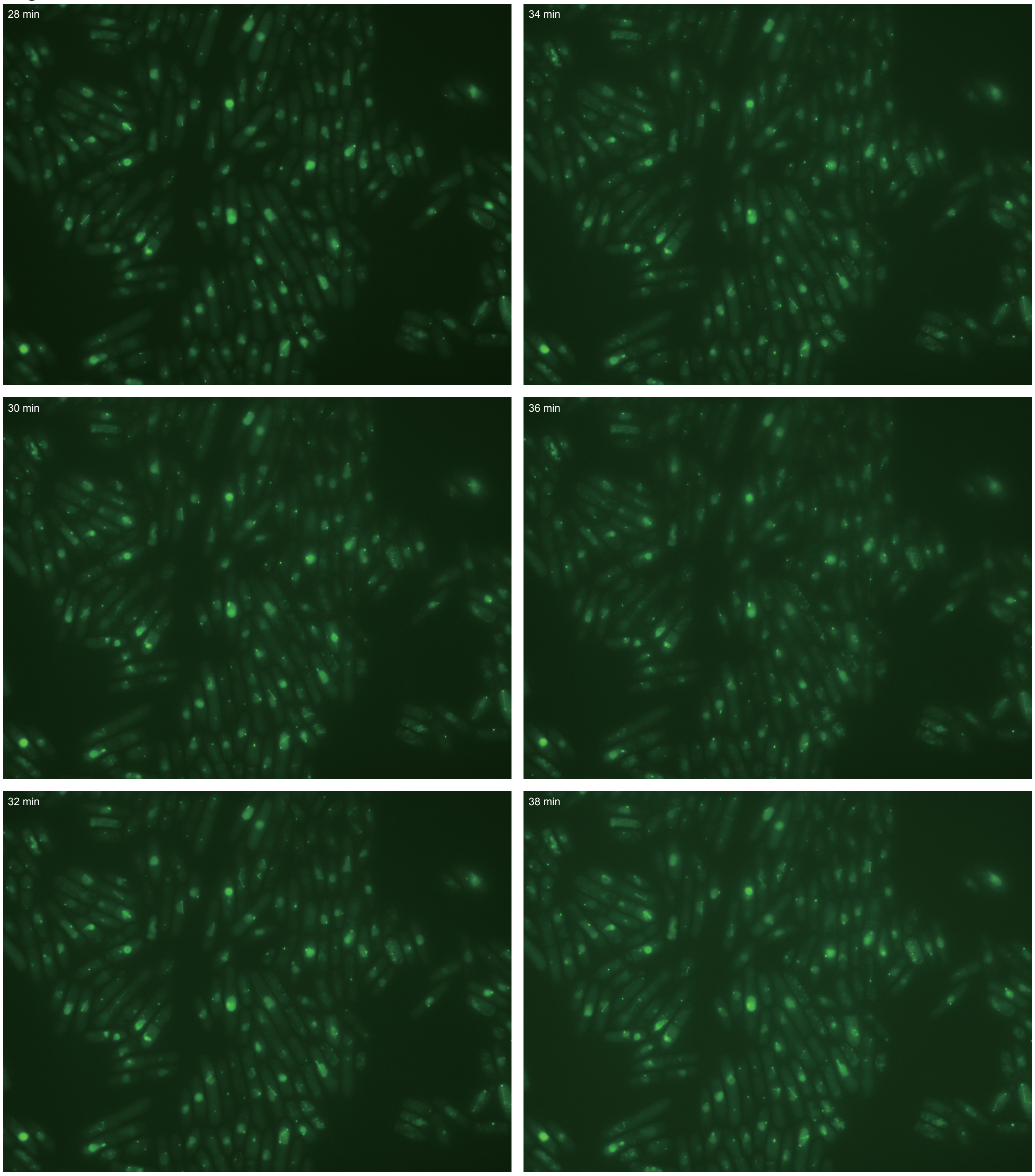

Figure 5E

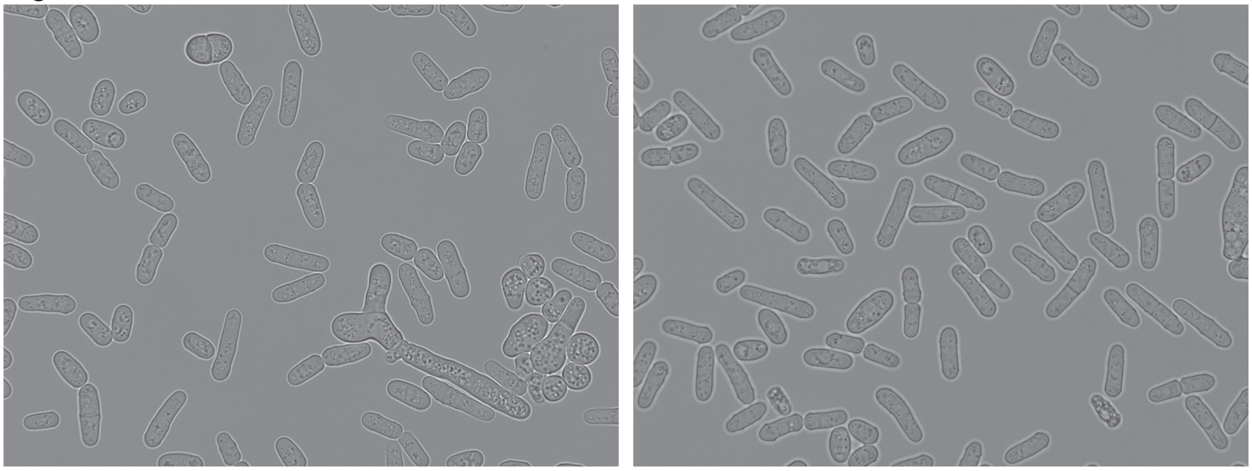

Figure 5E

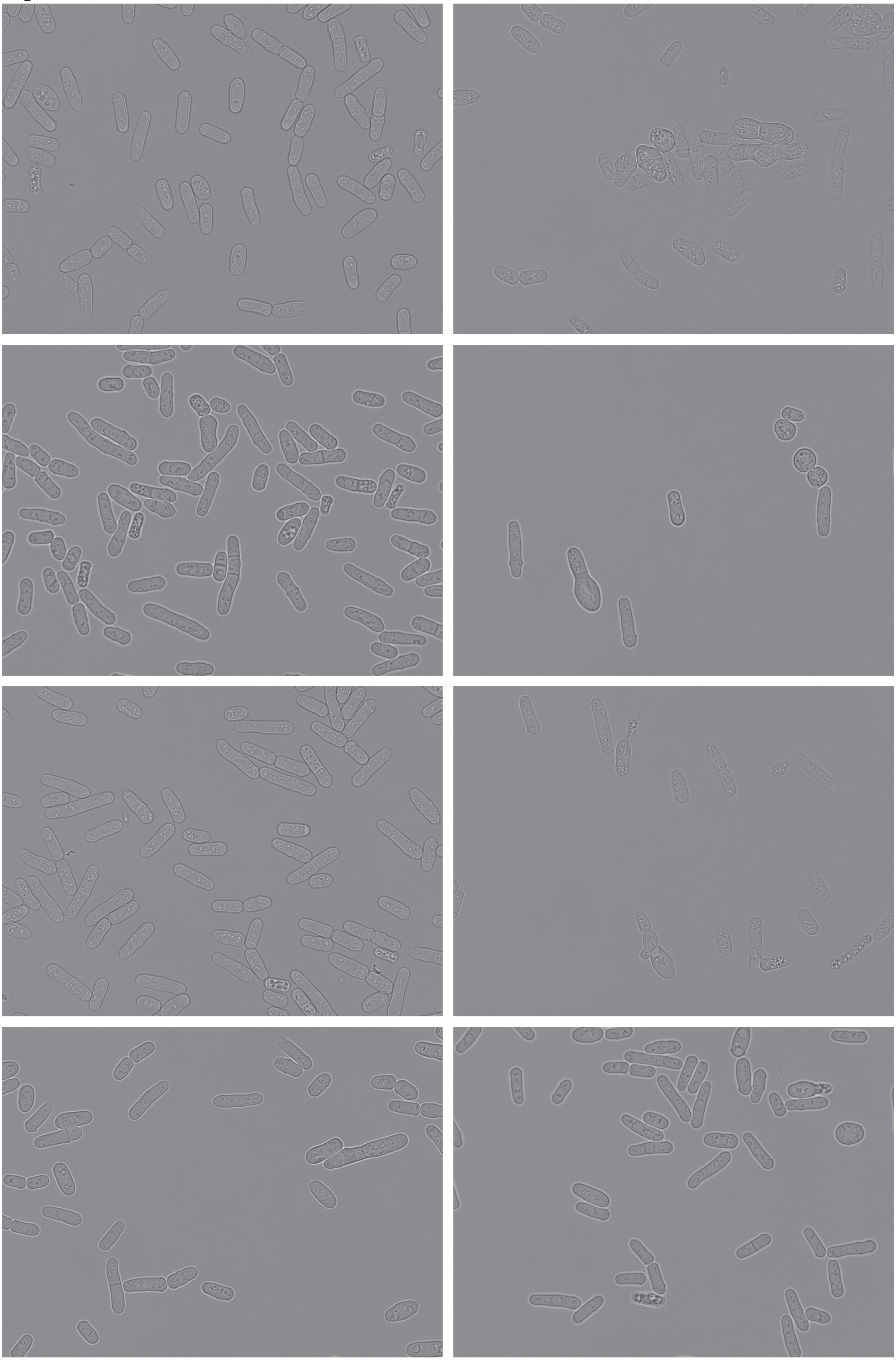

Figure 5E

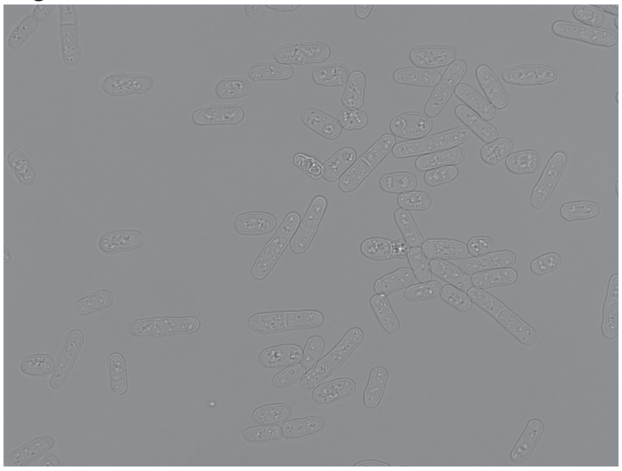

Figure 5D

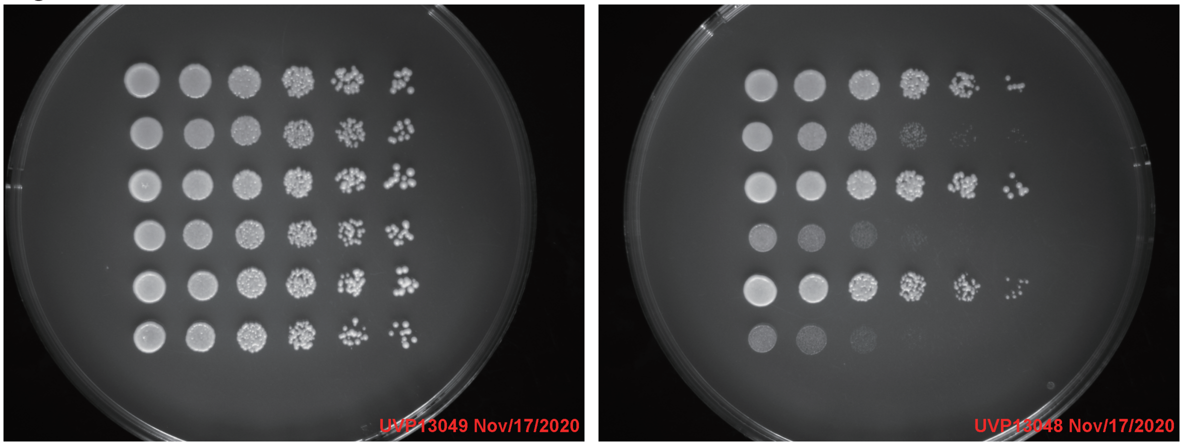

Supplement: Supplementary file 7 [file LSA-2022-01603_SdataF5.pdf]
