# Supplementary material for: Aberrant association of chromatin with nuclear periphery induced by Rif1 leads to mitotic defect
Source: Life Sci Alliance. 2023 Feb 7;6(4):e202201603. doi: 10.26508/lsa.202201603 (PMC9909590; doi:10.26508/lsa.202201603)

Kanoh *et al.* SourceData Supplementary Figure 5

Supplementary Figure 5B

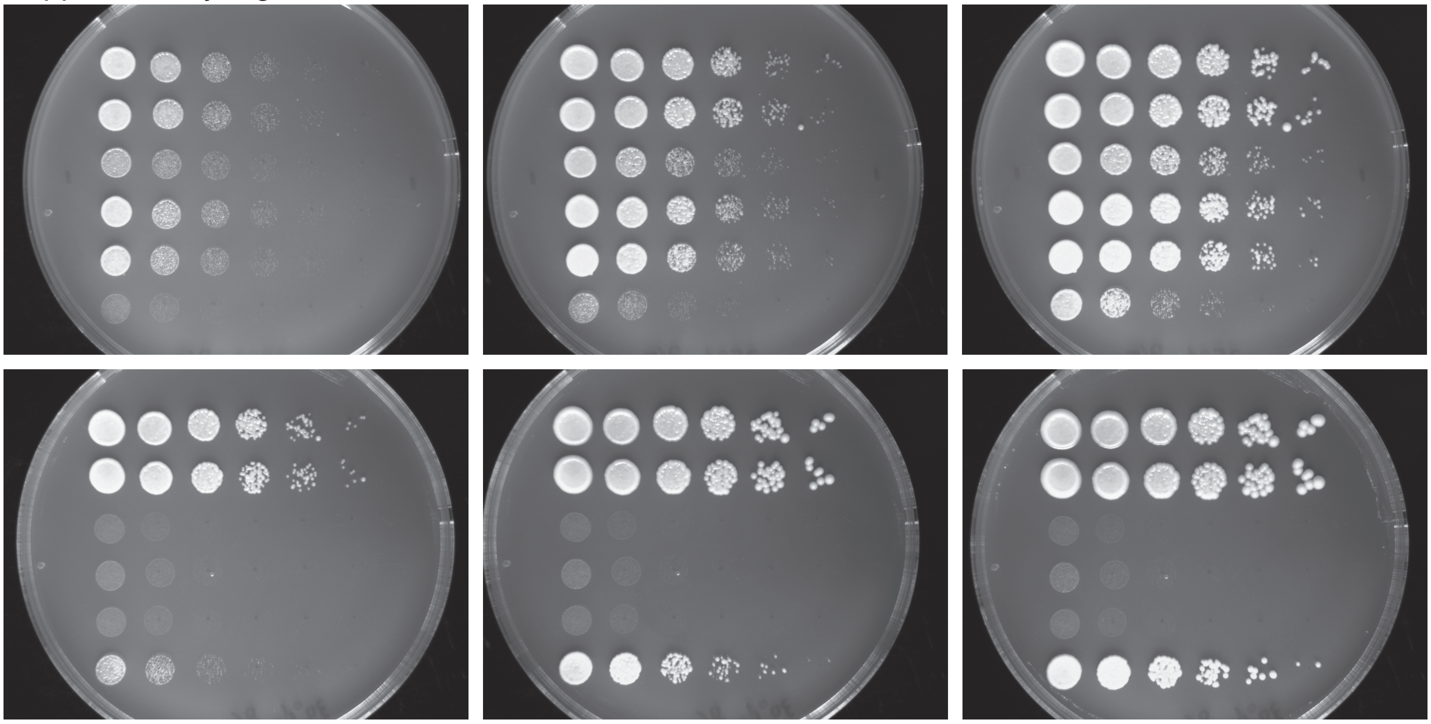

Supplementary Figure 5C

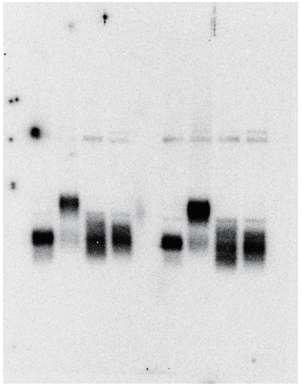

Supplementary Figure 5D

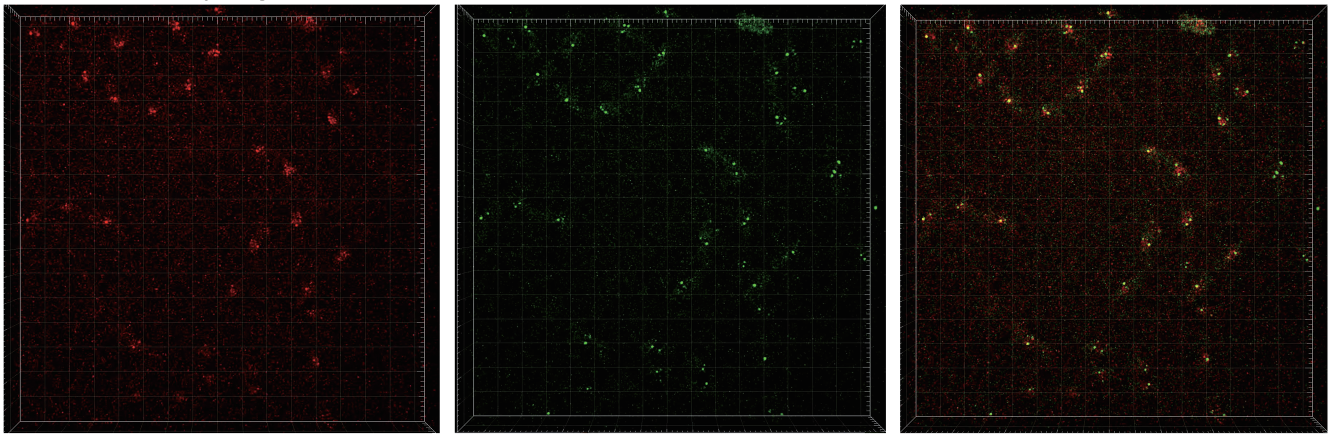

Supplementary Figure 5E

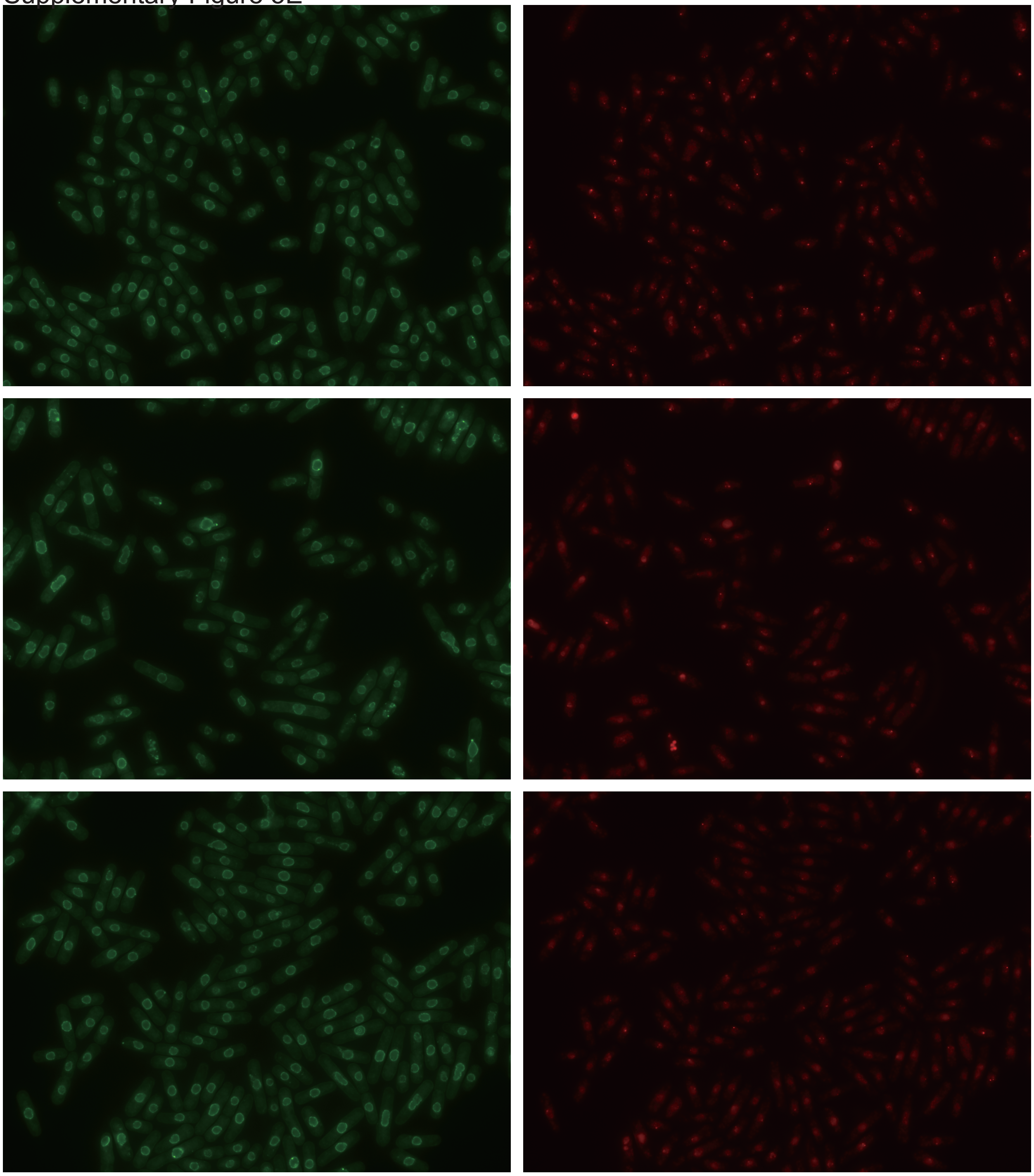

Supplement: Supplementary file 12 [file LSA-2022-01603_SdataFS5.pdf]
