# Supplementary material for: Aberrant association of chromatin with nuclear periphery induced by Rif1 leads to mitotic defect
Source: Life Sci Alliance. 2023 Feb 7;6(4):e202201603. doi: 10.26508/lsa.202201603 (PMC9909590; doi:10.26508/lsa.202201603)

Kanoh *et al.* SourceData Supplementary Figure 4

Supplementary Figure 6A Vector 1st

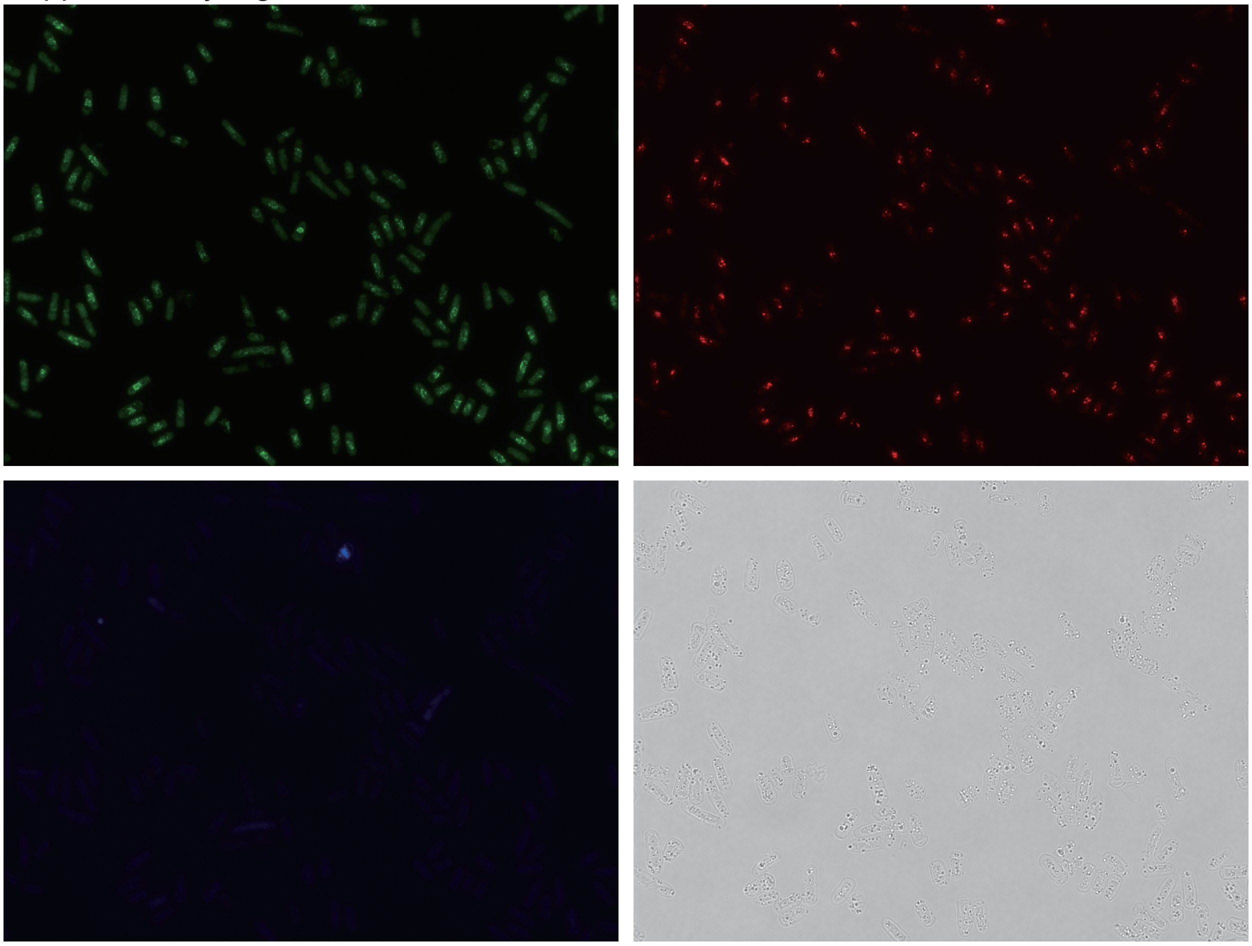

Supplementary Figure 6A Vector 2nd

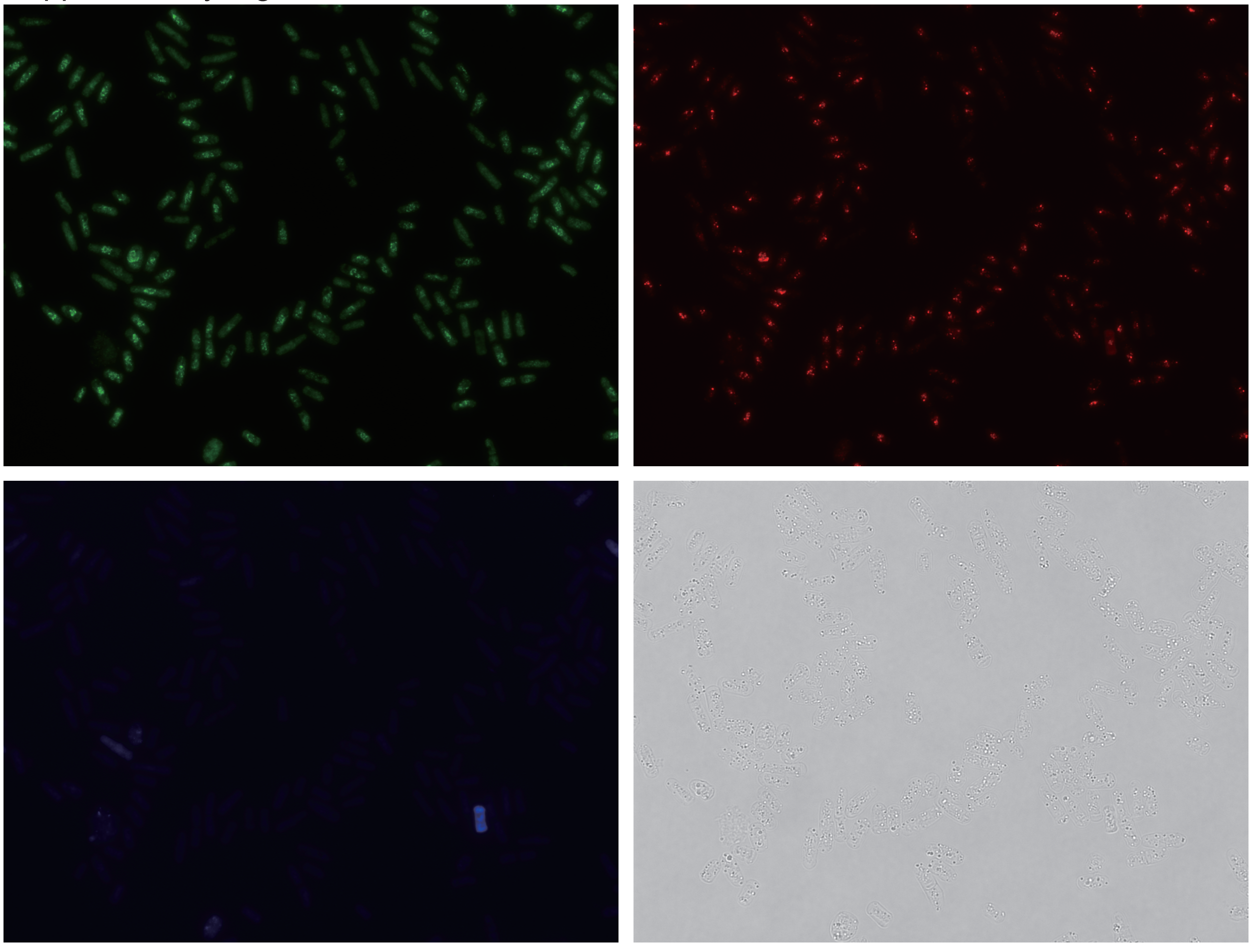

Supplementary Figure 6A Vector 3rd

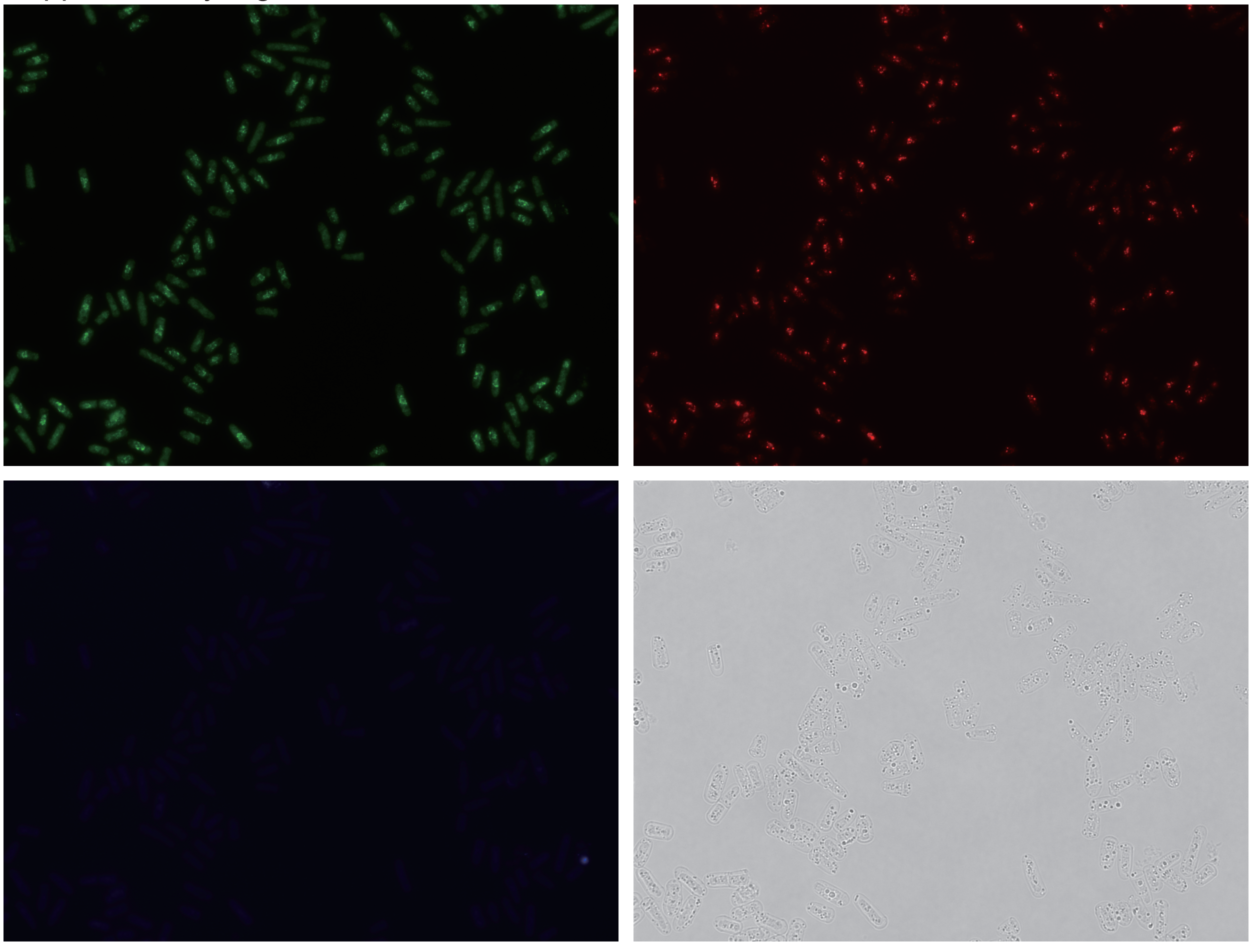

Supplementary Figure 6A Vector 4th

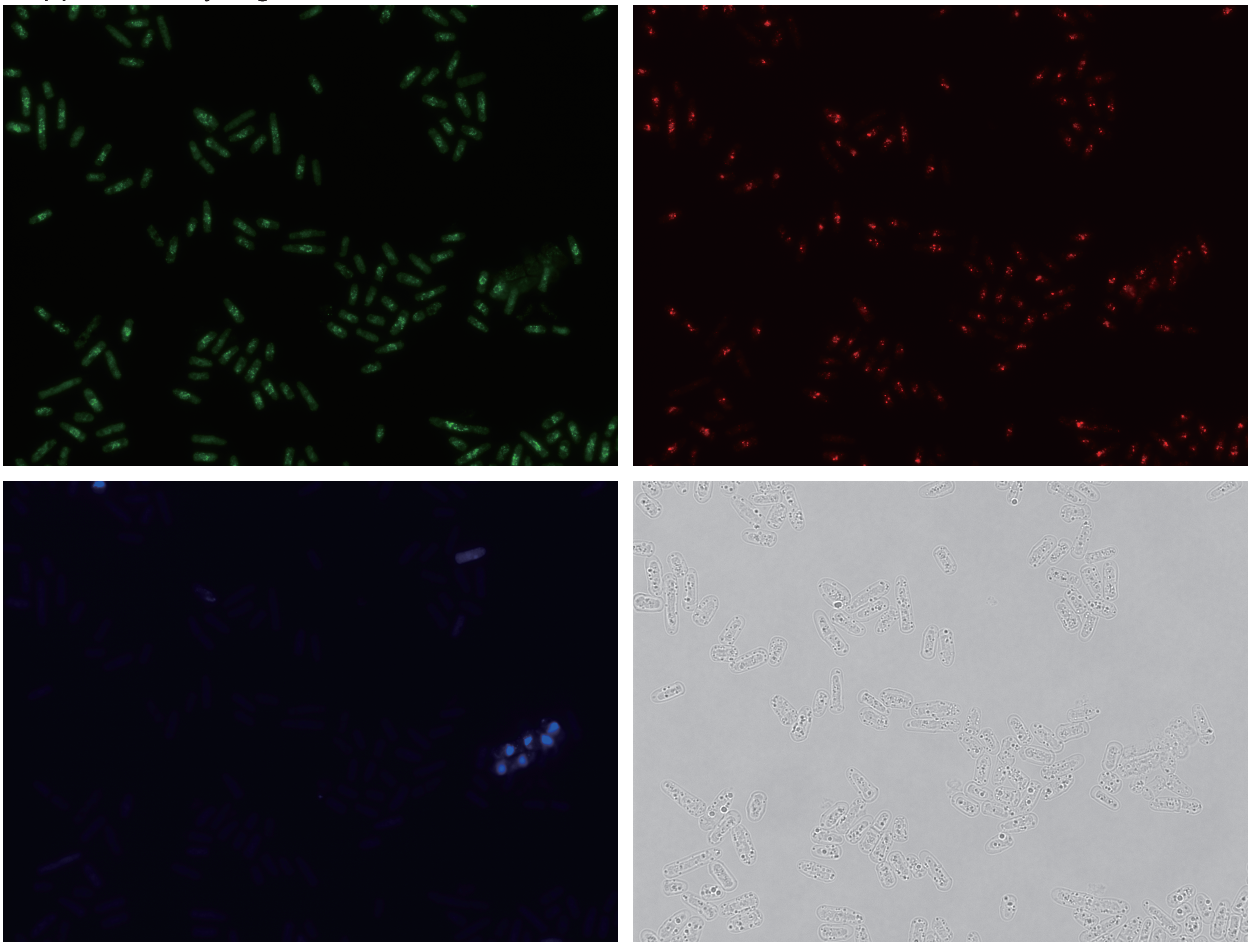

Supplementary Figure 6B Rif1 OE 1st&2nd

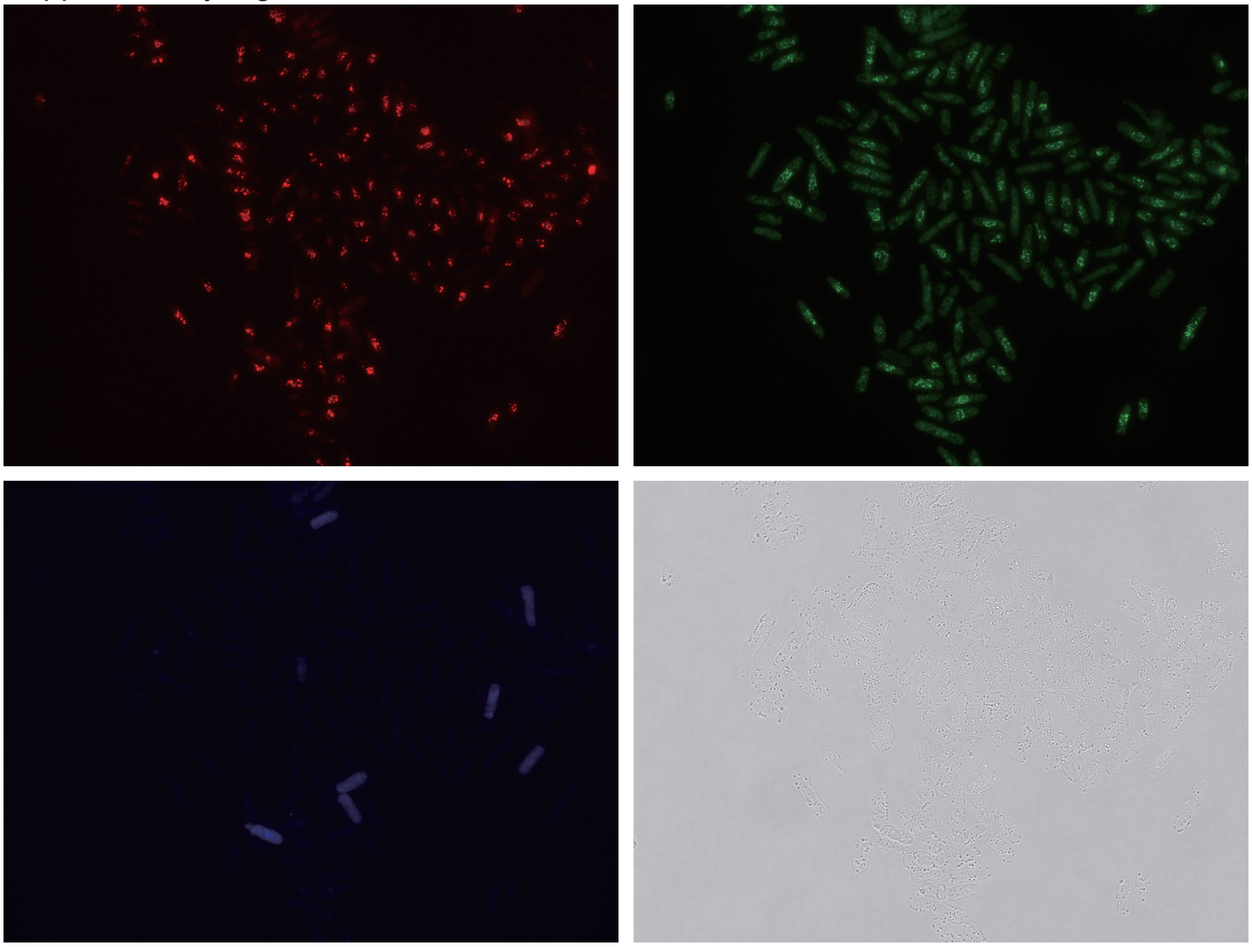

Supplementary Figure 6B Vector 3rd

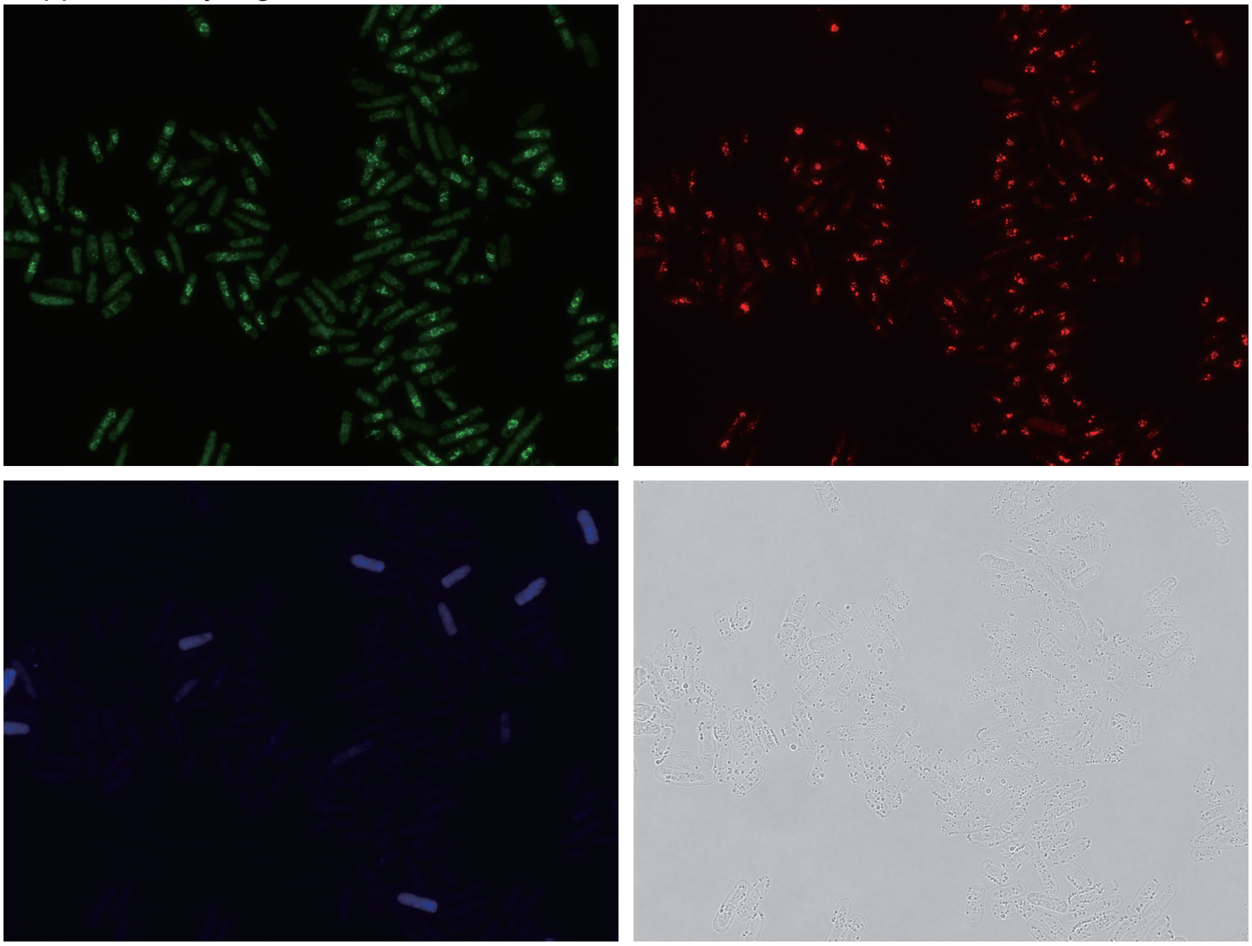

Supplementary Figure 6B Rif1 OE 4th

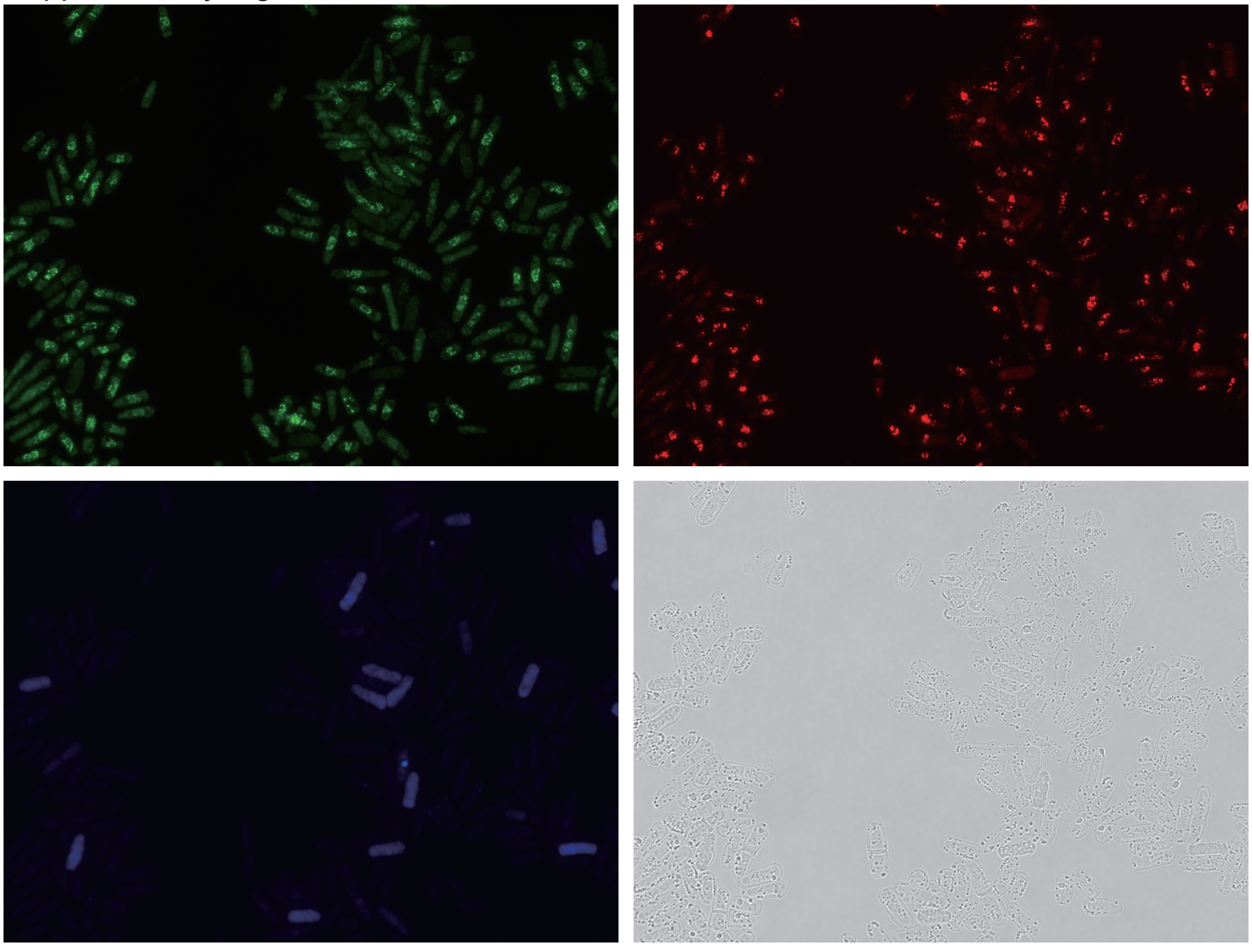

Supplementary Figure 6C Rif1 OE 4th

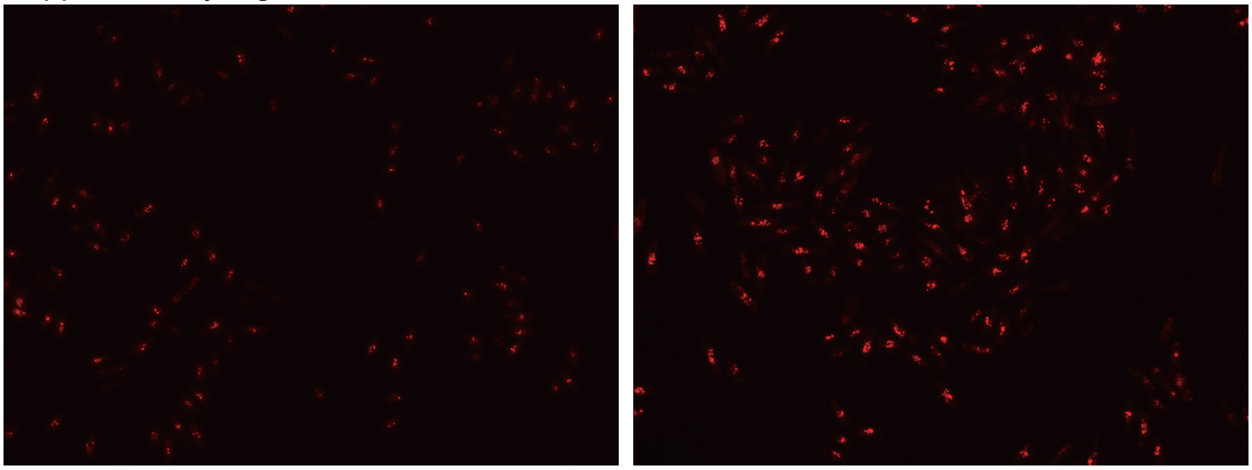

Supplement: Supplementary file 19 [file LSA-2022-01603_SdataFS6.pdf]
